# Production and characterization of magnetic Biochar derived from pyrolysis of waste areca nut husk for removal of methylene blue dye from wastewater

**DOI:** 10.1038/s41598-025-03359-z

**Published:** 2025-07-02

**Authors:** Syeda Minnat Chistie, Sneha Ullhas Naik, Pragathi Rajendra, Apeksha, Ranjeet Kumar Mishra, Gadah Albasher, Sampath Chinnam, Gautham P. Jeppu, Zeenat Arif, Javaria Hameed

**Affiliations:** 1 Department of Chemical Engineering, Ramaiah Institute of Technology Bangalore, Karnataka, 560054 India; 2 Department of Chemical Engineering, Manipal Institute of Technology, Manipal Academy of Higher Education, Manipal, 576104 Karnataka India; 3 Department of Zoology, College of Science, King Saud University, Riyadh, 11451 Saudi Arabia; 4 Department of Chemistry, Ramaiah Institute of Technology, Bengaluru, 560054 Karnataka India; 5 Chemical Engineering Department, Harcourt Butler Technical University, Kanpur, 208002 India; 6 Department of Building Civil and Environmental Engineering, Concordia University, Montreal, H3G1M8 Canada

**Keywords:** Adsorption, Biomass, Characterisation, Magnetic Biochar, Pyrolysis, Wastewater, Chemical engineering, Energy infrastructure, Environmental sciences

## Abstract

The textile industry causes lots of pollution due to its discharge of untreated coloured effluents into water bodies, impacting the environment. The present study includes a slow pyrolysis technique to produce magnetic biochar derived from waste areca nut husk (ANH)) biomass to adsorb methylene blue dye. The biochar and biomass were characterised via proximate analysis, ultimate analysis, bulk density, heating value, extractive content, biochemical analysis, thermogravimetric analysis (TGA), Fourier transform infrared spectroscopy (FTIR), SEM, BET surface area, pH, water holding capacity (WHC) and X-ray diffraction (XRD). A semi-batch reactor was used to produce biochar (ANHB) at 600 and 800 ^o^C at 10 ^o^C min^− 1^ heating rate and 45 min holding time in an inert atmosphere. The produced biochar was magnetised by blending aqueous biochar suspensions with aqueous Fe^3+^/Fe^2+^ solutions. Further, magnetised biochar is employed to eliminate methylene blue (MB) dyes at different pHs, contact times, temperatures, dosages and concentrations. Biochar derived at 800 ^o^C (ANHB800) gave increased carbon content (62.93%), heating value (33.02 MJ/kg), and BET surface area (112 m^2^/g) over biochar derived at 600 ^o^C. The results of the acid treatment biochar (ANHBA800) demonstrated that 5M H_2_SO_4_ causes a BET surface area increase (265 m^2^/g) and a ash content decrease (9.96%). However, when magnetic biochar was produced at 800 ^o^C it shows an additional increase in BET surface area upto 385 m^2^/g. The MB dye absorption analysis confirmed 85.47% adsorption at 0.3 g/l dosage, 100 ppm concentration, 30 ^o^C, 60 min contact time, and pH 7. The adsorption capacity was 785.34 mg/g when fit by the Langmuir isotherm model. Magnetic nanoparticles enhance active sites, electrostatic interactions, and recovery, improving efficiency, cost-effectiveness, and sustainability in dye removal. The adsorption kinetics results suggested that the pseudo-second-order model best explains the experimental data with an R^2^ value of 0.994. Additionally, the adsorption isotherm studies were best fitted by the Langmuir model adsorption conforming monolayer adsorption of MB on biochar surface.

## Introduction

The major causes of the dramatic increase in water pollution are the expanding human population, generation of hazardous waste, and discharge of raw wastewater from homes and industries^1^. Contaminated water bodies pose significant threats not only to marine and aquatic life but also to human health and well-being. Depending on their source, pollutants released into water bodies can generally be categorised as organic, inorganic, or radioactive. Industries such as textile, leather, paper, rubber, food processing, printing, and cosmetics are among the primary contributors to water pollution. These water bodies become polluted when industrial effluents are discharged without undergoing proper treatment^2,3^. Among these industries, the textile sector is particularly concerning as it is the largest contributor of untreated coloured effluents into water bodies and has a high water consumption for dyeing processes. Approximately 1–2% of dyes during production and 1–10% during utilisation are estimated to be released into effluents^4^. The harmful nature of these dyes, even in small concentrations, can be attributed to their highly complex structures, which make them challenging to decolourise^4,5^. Furthermore, organic dyes are the most common environmental contaminants and are utilised extensively in a variety of sectors, including the production of paper, leather, textiles, and medicine. Human health hazards from these dyes include nausea, high blood pressure, mental difficulties, and stomach pain. In textile companies, methylene blue (MB) dye is a cationic dye that is frequently used for a variety of purposes, such as coating leather, colouring paper, silk, wool, and cotton^6^. People and aquatic animals may suffer irreversible harm from eye burns brought on by MB exposure^7^. Human ingestion can cause symptoms such *nausea*,* vomiting*, *diarrhea*, *gastrointestinal distress*,* erratic heartbeat*,* mental disorientation*, and shock^7^. The removal of MB from industrial effluents is a significant environmental concern since cationic dyes are typically more harmful than anionic dyes^8,9^.

In order to properly treat industrial effluents, these difficulties have prompted the development of sophisticated water treatment methods, including membrane filtration, flocculation, biological treatments, electrolysis, and oxidation. Despite their efficiency in removing dyes, these methods face limitations, including high initial investment, operational costs, and challenges in managing secondary sludge^8–10^. The adsorption process is considered the most effective method for removing dye molecules from wastewater due to its numerous advantages, including a simple design, ease of operation, large surface area, high adsorption efficiency, chemical stability, environmental friendliness, and cost-effectiveness^7^. Various adsorbents, such as activated carbon and biochar derived from waste materials, have been extensively studied for this purpose. Biochar or activated carbon (AC) has garnered significant attention from the scientific community for its potential for environmental remediation. Activated carbon (AC) has been the most widely used adsorbent across the world. However, the AC has many shortcomings that limit its application in water treatment, such as high cost, a complicated separation procedure, and complex regeneration^11^. The decomposition of biomass thermally under oxygen-deprived circumstances is known as biochar. Biomass is converted using a variety of techniques into carbon-rich microporous materials with a high degree of aromatisation and a well-developed porous framework^12^.

The production of magnetic biochar adsorbents for the treatment of water and wastewater is becoming increasingly popular. Hazardous to human health and the environment are toxic pollutants like lead and cadmium, which are primarily found in industrial waste. Magnetic bio-sorbents derived from organic and renewable resources are emerging as novel adsorbing materials for environmental remediation due to their affordability, accessibility, and environmental friendliness^11^. Low-intensity external magnetic fields may be used to manage magnetic adsorbents, making it possible to recover polluted water with a high concentration of suspended particles, oil, and grease^13^. The contaminants may result in carbon fouling and necessitate repeated regeneration or separation procedures in definite situations. Magnetic separation simplifies re-dispersion after washing and isolation. The biochar is magnetised and effectively applied for the remediation of various pollutants in water under varying temperatures, pH levels, and solid-to-liquid ratios^13^. Studies have shown that biochar often outperforms other materials in adsorption efficiency, making it a promising solution for wastewater treatment^14,15^. One of the most outstanding properties of biochar is its ability to reduce the bioavailability of contaminants, preventing their transfer from water to organisms through adsorption and partitioning. This is complemented by its high porosity, graphene-like carbon matrix, enhanced surface functional groups, and improved ion exchange capacity. Although a variety of waste materials can be converted into biochar for adsorption purposes, key challenges include high production costs and limited substrate regeneration capacity. To overcome these obstacles, researchers are turning their attention to adsorbents derived from low-cost and readily available biomass.

Various materials, including algal biochar, maize cobs, stover, orange peels, sludge, dairy manure, agricultural residues, and forestry waste, have been utilised to produce biochar, which is subsequently modified through chemical and physical treatment^16,17^. Zheng et al. (2023) examined the adsorption capacities of modified biochar for removing sulfamethylimidine and methylene blue (MB) from water. The biochar was modified to enhance its surface properties, resulting in improved adsorption performance^18^. Sawalha et al. (2022) studied MB adsorption using biochar from various agricultural residues, including coffee grains, almond shells, and peanut hulls. Biochar, prepared via pyrolysis and activated with ZnCl₂, showed over 95% MB removal efficiency for peanut hulls, with sunflower shells being the most effective after activation^19^. Hoslett et al. (2020) produced biochar from mixed municipal waste at 500 ^o^C using a heat pipe reactor to eliminate MB dye. The biochar exhibited a maximum adsorption capacity of 7.20 mg/g at an initial MB concentration of 100 mg/L. Adsorption followed the Langmuir isotherm, suggesting monolayer adsorption, with π-π interactions and electrostatic attraction recognised as primary mechanisms^20^. Several other studies on the removal of methylene blue (MB) dye from wastewater and aqueous solutions are summarised in Table 1. The Scopus data search between 2010 and 2024 on keywords water and wastewater treatment on 06/02/2024 showed 22,193 research articles, 194,058 research articles, 15,111 book chapters and others. However, a specific search on water and wastewater treatment using biochar shows 4026 review articles, 13,745 research articles, and 1550 book chapters. Further searching with keywords water and wastewater treatment using magnetic biochar 2052 review article, 5727 research articles, and 569 book chapters. After reviewing several pieces of literature, it was found that most research utilises areca nut husk to produce renewable fuel and valuable chemicals. Furthermore, only a few research concentrated on employing hydrothermal carbonization to produce hydrochar for soil-related applications. In-depth research on the production and characterization of biochar through slow pyrolysis of areca nut husk (ANH) is quite limited. To the best of the author’s knowledge, no studies have been conducted on the treatment of MB dyes using magnetic biochar derived from ANH. This study uniquely develops magnetic biochar from areca nut husk via slow pyrolysis for methylene blue dye removal, enhancing adsorption efficiency and reusability. Unlike previous studies, it integrates magnetization for easy separation, optimises acid treatment for surface enhancement, and systematically evaluates adsorption-desorption cycles.

In light of the aforementioned research gap, the present study is focused on the production, characterisation of magnetic biochar and treatment of MB dyes in wastewater using Uv-Vis spectroscopy. ANH was characterised via proximate analysis, ultimate analysis, bulk density, heating value extractive analysis, biochemical analysis, thermogravimetric analysis (TGA), Fourier transform infrared spectroscopy (FTIR), and X-ray diffraction (XRD). Further, magnetic biochar was characterised via proximate analysis, ultimate analysis, TGA, FTIR, XRD, FESEM, BET surface area, pH, heating value, water holding capacity (WHC), etc. The resulting biochar is then tested in batch adsorption studies to remove methylene blue (a cationic dye) from aqueous solutions. To determine the optimal operating conditions for maximum removal efficiency, adsorption experiments are conducted with fluctuating constraints such as doses (0.1–1 g), initial dye concentration (25–125 mg/L), contact time (15–120 min), temperature (25–50 °C), and pH (2–12). The adsorption mechanism is analysed using isotherm and kinetic studies, employing two types of isotherms and four different kinetic models.

**Table 1:** List of literature published on the removal of MB dye using Biochar or Biochar-based material.

Biochar	Treatment	Initial dose (g)	pH	Content time (min)	Temperature (°C)	% removal	Adsorption capacity (mg/g)	References
Chlorella vulgaris microalgal biochar	–	3	7	60	30	85.74		^21^
Lignin-derived porous biochar	KMnO_4_, MnSO_4_, and MnO_2_	20 mg	2	60	30	99.73	248.96	^22^
Rice husk biochar (RHB), cow dung biochar (CDB) and domestic sludge biochar (SB)	–	0.5–0.6 mg/l	2–11	5 dyes	–	7.0–99.0; 71–99 and 73-98.90%	–	^23^
Empty fruit bunch biochar	Magnetic biochar	1	2–10	120	30	–	31.25	^24^
Sugarcane bagasse Biochar	–	–	4.5	360	30	–	17.20	^25^
Cattle manure-derived biochar		0.01–0.5 g	3–4		25–35	–	241.99	^26^
Pine wood (BC-PW), pig manure (BC-PM) and cardboard (BC-PD) biochar	Microparticle	25 mg	–	–	25	–	25 ± 1.3	^27^
Walnut shell (WSC) and wood powder biochar (WPC)	ZnCl_2_, KOH, H_2_SO_4_, and H_3_PO_4_	20, 30, 40, 50, 80, and 100 mg/l	–	240	30	–	701.30 and 850.90	^28^
Date Palm Fronds waste biochar	–	200 mg/L	6	300	30	–	206.61	^29^
Areca nut husk biochar	H_2_SO_4_, Magnetic biochar	3	7	30	30	89	785.34	Present study

## Materials and methods

### Sample collection and Preparation

Areca nut husk (ANH) is sourced from a nearby town in Bangalore, Karnataka, India. The biomass was chopped into smaller pieces (2–5 cm) and oven-dried at 105 ^o^C for 24–36 h to remove humidity uniformly. Further, ANH was then ground into the required particle size (1–2 mm) after being oven-dried. The pulverised biomass was then sealed within an airtight plastic container to protect it from moisture.

### Chemical and reagents

Analytical-grade chemicals with 99% purity were utilised throughout. Ferric sulphate (> 98%), ferrous sulphate (99.50%), Hydrochloric acid, and sodium hydroxide (99%) were bought from Merck, India. Methylene blue dyes (C_37_H_27_N_3_Na_2_O_9_S_3_) and H_2_SO_4_ were purchased from Sigma-Aldrich from Sigma-Aldrich, Mumbai, India.

### Physicochemical characterisation of biomass

Proximate analysis is the first level of the characterisation approach of feeds, which indicates moisture content (MC), volatile matter (VM), ash content (A), and fixed carbon (FC). Ash and moisture content were calculated using ASTM D 2974-8, whereas volatile matter was calculated using ASTM D 4559-99. Additionally, the ultimate analysis was carried out using a CHNS/O elemental analyser (Variael dice, Germany). The size, shape, density, moisture content, surface properties, and degree of tightness of the biomass all influence its bulk density. Also, the transportation and storage of biomass are intimately correlated with its bulk density. Therefore, the bulk density of the biomass was determined using a digital scale and graduated cylinder. A computerised weighing scale was used to determine the sample’s weight, and a graduated cylinder was used to determine its volume. The higher heating value (HHV) of the sample is determined by combusting solid fuel in a controlled environment using an oxygen-bomb calorimeter (Parr 1108P). Additionally, the extractive content of ANH was assessed using a Soxhlet device with hexane and ethanol solvents. Finally, the Van Soest techniques were used to identify the biochemical constituents of biomass^30^.

### Thermal analysis

Thermogravimetric analysis (TGA, STA449, NETZSCH) is used to conduct a thermal analysis of the material (biomass and biochar). In an aluminium crucible with nitrogen gas flowing continuously at a rate of 50 mL min^− 1^, 8.0 mg of sample was heated from 30 to 900 °C. The weight loss of the biomass against temperature was plotted, and the resulting data was examined utilising origin software ( OriginPro 2025, 10.2) .

### FTIR analysis

Fourier transform infrared spectroscopy (FTS 3500 GX used in conjunction with DRS) has been used to classify the functional groups of biomass and biochar. In a sample container, a small quantity of material was mixed 1:100 with oven-dried potassium bromide (KBr) powder and placed in the 400–4000 cm^− 1^ wavenumber range at a scanning rate of 40 and a step size of 4 cm^− 1^.

### X-ray diffraction (XRD) analysis

The Rigaku TT Rax diffractometer was used in conjunction with a Cu-K radiation source, and X-rays were produced at 9.0 kW and 250 mA to acquire the XRD pattern of the samples. All samples were scanned at an angle of 5° to 50° per second (0.03 min^− 1^). The equation below was used to determine the crystallinity index of biomass samples:

1 $$Crl\,(\% )=\left[ {\frac{{Crystaline - Amorphoues}}{{Amorphous}}} \right] \times 100$$

### Biochar production

**Fig. 1:**
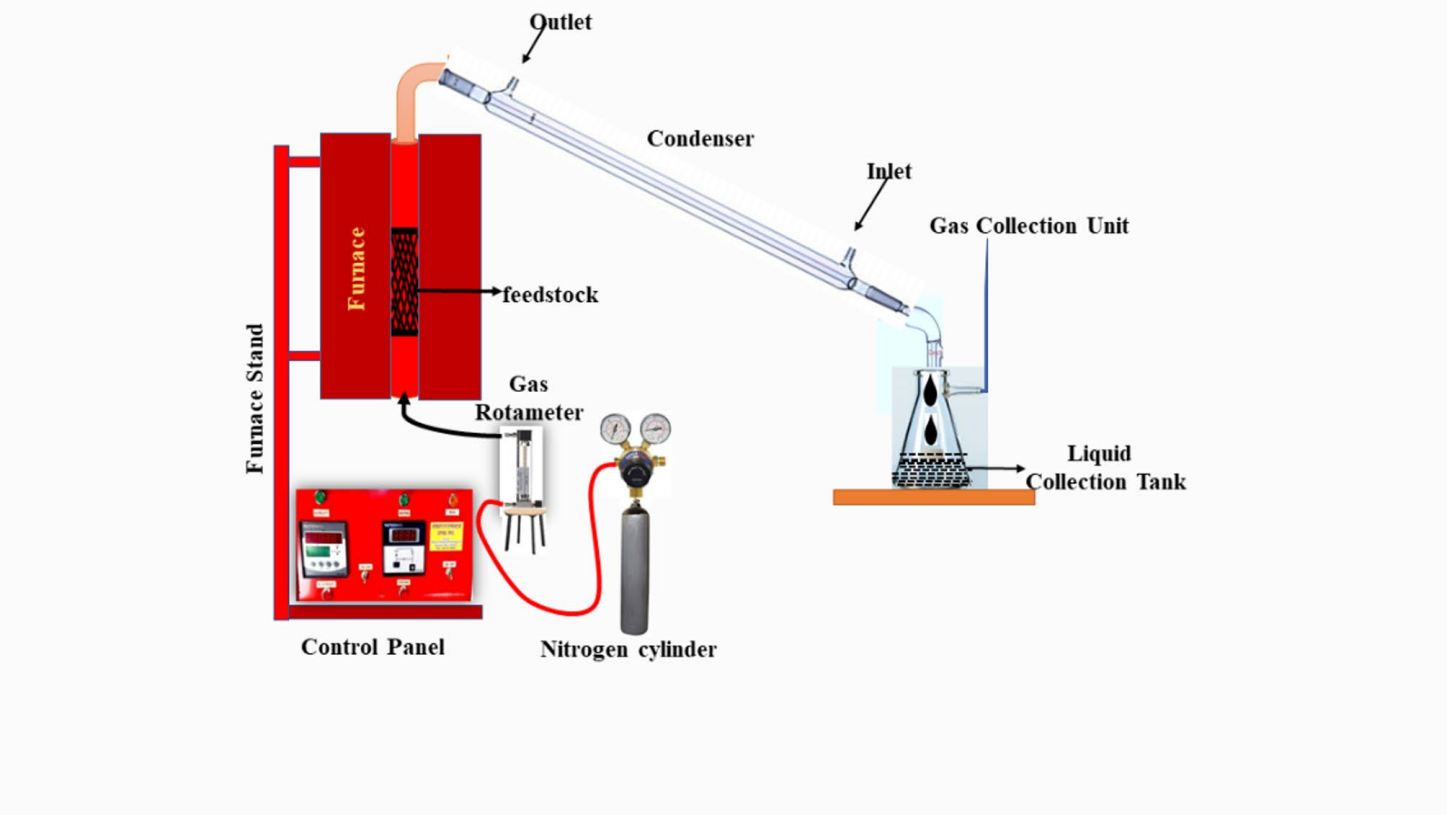
Schematic presentation of the experimental setup.

A stainless steel (SS-304) cylindrical semi-batch reactor with dimensions of 8 cm x 8.4 cm and 40 cm in length was used to generate the biochar. The pyrolysis test was conducted at 600 and 800 ^o^C with a 45 min holding time in a nitrogen environment. The holding time is the amount of time needed for the material to decompose as much as possible during the pyrolysis process at a particular temperature. Throughout the experiment, the heating rate was held constant at 10 ^o^C min^− 1^. A ceramic block was used within the furnace to stop heat loss, and the experimental setup’s outside was composed of stainless steel. Additionally, 500 g of oven-dried dry biomass was placed vertically inside the reactor. A thermocouple, control panel, quartz reactor, gas rotameter, nitrogen cylinder, and conical flask are the primary components of pyrolysis reactors. The control panels establish the required temperature, holding time, and residence time based on the thermocouple, which is directly connected to the furnace and keeps track of the precise temperature inside. The gas rotameter was used to measure the nitrogen gas flow rate precisely. The nitrogen gas was begun 15 min prior to the test in order to remove any unwanted gasses and pollutants from the reactor. However, it was let to flow continuously at a rate of 1.5 L per minute (LPM) throughout the test. To ensure that the biochar yield was accurate, the pyrolysis test was performed three times. Once the test was completed, the reactor was allowed to cool to 30 ^o^C. An airtight plastic container was used to store the solid residual product from the experiment. The biochar yield was computed using Eq. (2). A lab cum mixing grinder was utilised to break down the biochar into smaller particle sizes (500–700 nm) for more research. The experimental setup is schematically shown in detail in Fig. 1.

2 $$\% \,\,Yield\,of\,biochar=\left[ {\frac{{Solid\,material\,left\,after\,the\,pyrolysis}}{{Weight\,of\,feeded\,biomass}}} \right] \times 100$$

### Characterization of Biochar

The methods stated in **Sect. 2.2** (Physicochemical characterisation of biomass) for analysing biochar included proximate analysis, ultimate analysis, HHV, bulk density, TGA, FTIR, and XRD. Further, dry biochar was used to estimate BET surface area using N_2_ adsorption at 77 K on a BET Surface Area Analyzer (Micromeritics ASAP-2020 BET). The biochar was degasification at 250 ^o^C for 5 h to eliminate the moisture. The pH of the biochar was determined by calibrating with standard pH buffers at pH 4, 7, and 10. Further, with the use of a Buchner funnel, distilled water, and filter paper, the water holding capacity (WHC) of biochar was calculated. A Buchner funnel-lined filter was carefully loaded with 1 g of biochar. The filter paper (Whatman no. 5) is equally coated with the sample, which has also been precisely sprayed with water. To reduce evaporation loss, the funnel was covered from the top, and samples were let to drain freely until every last drop departed. Weighing the wet biochar, subtracting the known weight of the dry sample, and accounting for the weight of the filter paper determine the biochar’s ability to hold water. Further, an Energy Dispersive X-ray Spectrometer (EDS, Oxford Inca Energy 350) and Field Emission Scanning Electron Microscope (FESEM, Zeiss Supra 40) were used to analyse the morphology of biochar. To avoid sample charging, the double gold coating was applied to the biochar, which was placed over a piece of carbon tape. With acceleration voltage ranging from 5 kV to 15 kV, FESEM was employed in a high vacuum environment. Three replicas of each analysis were run, and the average findings are presented in this paper. Magnetic characterisation was conducted using a Lake Shore 7304 Vibrating Sample Magnetometer (VSM). The magnetic properties of biochar were analysed by plotting magnetization (M) against the applied magnetic field (H), generating hysteresis loops. The saturation magnetization values were obtained from these loops to assess magnetic strength.

### Acid pre-treatment of Biochar

The biochar produced from the thermochemical process encompasses high ash content, which limits the application of biochar; thus, elimination of the ash content becomes essential. The chemical treatment of biochar was found to be an efficient method of removing ash content from biochar compared to the physical treatment^31^. Acid treatment involves soaking biochar in an acid solution, H_2_SO4, to remove the ash content. The stock solution was prepared at 1 M, 5 M, 7 M and 9 M of H_2_SO_4_, respectively, in 100 mL distilled water. Further, 2 g of biochar was added to each beaker and a stirrer for 3 h. The solution was filtered using Whitman filter paper and Buchner funnel by repeatedly flushing 4–5 times with distilled water, followed by washing with ethanol to remove acidity. The acid-treated biochar was then oven-dried for 3 h and stored in an air-tight glass jar.

### Preparation of magnetic Biochar

**Fig. 2:**
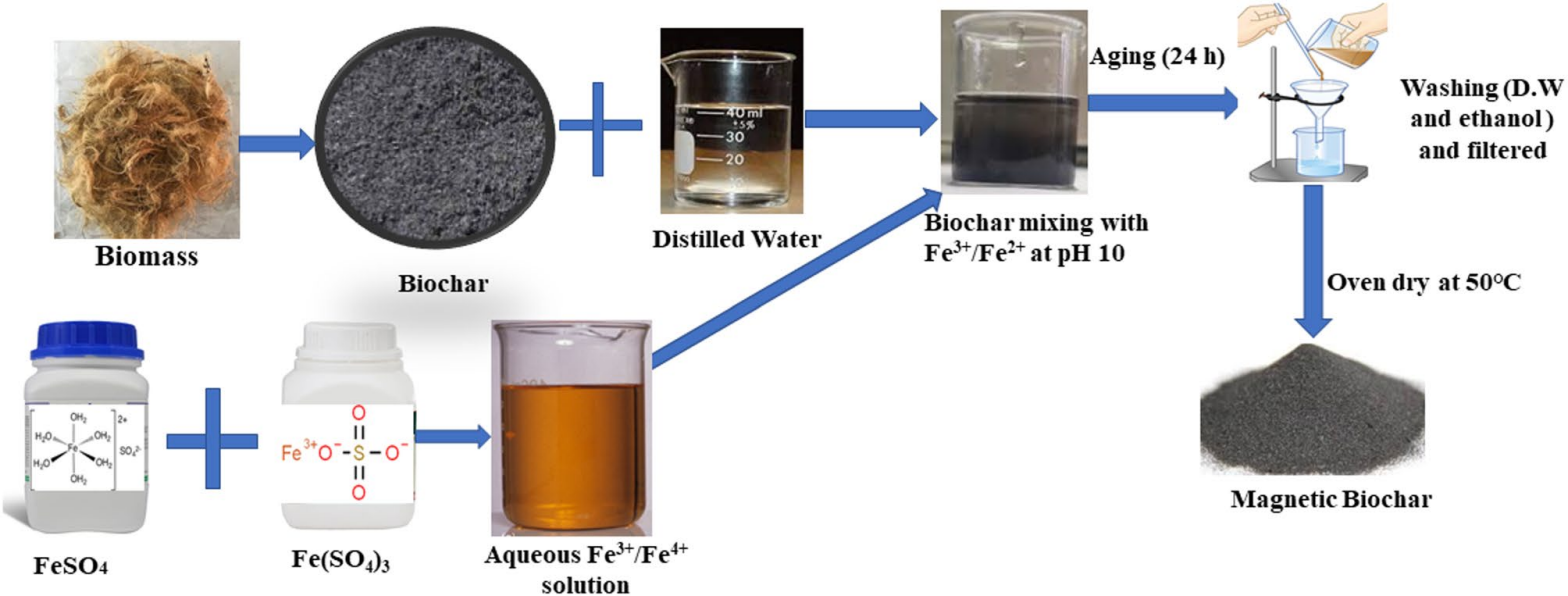
General scheme for the magnetic biochar preparation.

The biochar produced at 800℃ was modified into magnetic biochar, and a schematic layout of the procedure is shown in Fig. 2. The biochar produced at 800 ^o^C has greater properties than 600 ^o^C; thus, 800 ^o^C derived biochar was used for magnetic biochar production. A freshly prepared ferric sulfate solution was obtained by dissolving 18.50 g of Fe₂(SO₄)₃·nH₂O (where *n* = 6 to 9) in 1300 mL of distilled water. Separately, a ferrous sulfate solution was prepared by dissolving 20 g of FeSO₄ in 150 mL of distilled water. Fatretaht, both the solutions were thoroughly mixed and stirred at 60–70 °C. The resulting Fe²⁺/Fe³⁺ sulfate solution was introduced into an aqueous suspension containing 50 g of biochar at room temperature and gently stirred for 60 min. Furthermore, 10 M NaOH solution was gradually added dropwise to the biochar/Fe²⁺/Fe³⁺ suspension until the pH reached 10–11. During the NaOH addition, the suspension initially turned dark brown at pH 6 and gradually became black at pH 10. Concentrated NaOH was added to the iron suspension to facilitate the precipitation of iron hydroxides, which later transformed into magnetic iron oxides (e.g., Fe₃O₄). This process enhances the formation of magnetic biochar by ensuring uniform nanoparticle deposition, improving adsorption properties, and enabling easy magnetic separation in wastewater treatment applications. After continuous stirring for 60 min, the mixture was left undisturbed at room temperature for 24 h and then filtered. The collected filtrate was repeatedly washed with distilled water, followed by ethanol until it reached a neutral pH. The resulting magnetic biochar was then vacuum-filtered and dried overnight at 50 °C in a hot air oven.

### Adsorbate dye solution Preparation

Methylene blue (MB; Sigma-Aldrich, CAS: 61-73-4, C₁₆H₁₈ClN₃S, MW 319.90 g/mol) dye was selected for batch adsorption studies. A 1000 ppm stock solution was prepared by dissolving 1 g of MB in 1 L of distilled water, and the required concentrations were obtained through dilution. The solution pH was adjusted using 0.1 M HCl for acidic conditions and 0.1 M NaOH for alkaline conditions, with a pH meter calibrated against standard buffers (pH 4, 7, and 10) used for confirmation.

### Batch sorption experiments

Biochar was used in batch adsorption tests to remove methylene blue dye in a 250 mL conical flask that was kept in a water bath shaker with a continuous stirring speed of 100 rpm. The ideal conditions for optimum adsorption effectiveness were found by methodically varying key adsorption process variables, such as adsorbent dosage, reaction temperature, initial dye concentration, contact time, and mixture pH. The adsorbent dosage ranged from 0.1 to 1 g, the reaction temperature ranged from 30 to 50 °C, the starting dye concentration ranged from 10 to 200 mg/L, the contact period ranged from 30 to 120 min, and the pH ranged from 3 to 10. Every experiment was carried out three times, and the average results were shared. Normal sunlight conditions were used for the adsorption trials. Further, a UV-vis spectrophotometer was used to analyse the methylene blue dye content. Equation 3 was used to calculate the percentage removal of MB.

3 $$\:Removal\:\left(\%\right)=\frac{{C}_{o}-{C}_{e}}{{C}_{o}}\times\:100$$

Where C_0_ = initial dye concentration (mg/L) and C_e_= equilibrium dye concentration after the experiment (mg/L).

### Isotherm and kinetics studies

The adsorption performance for MB dye was achieved via Langmuir and Freundlich isotherm models, whereas adsorption kinetics was determined using pseudo-first-order, pseudo-second-order and Elovich models. The kinetics study and adsorption isotherms were calculated using MATLAB R2020 software, and the best-fitted value with experimental data was assessed via R^2^. The major model equations used in this study are listed in Table 2.

**Table 2:** Isotherm and kinetics models were used.

Model name	Equations
Langmuir	$\:{q}_{e}={q}_{m}{K}_{L}\frac{{C}_{e}}{1+{K}_{L}{C}_{e}}$ (4)
Freundlich	$\:{q}_{e}={K}_{F}{C}_{e}^{\frac{1}{n}}$ (5)
Kinetic model	
Pseudo-first order	$\:{q}_{e}={q}_{e}\:(1-\text{exp}(-{k}_{1}t))$ (6)
Pseudo-second order	$\:{q}_{e}=\frac{{q}_{e}^{2}{k}_{2}t}{1+{q}_{e}{k}_{2}t}$ (7)
Elovich	$\:{q}_{e}=\left(1+{\beta\:}_{E}\right)\times\:\text{l}\text{n}(1+{\alpha\:}_{E}{\beta\:}_{E}t)$ (8)

q_e_ and q_m_ = the amount of dye adsorbed at equilibrium and saturation (mg/g), C_e_ = equilibrium dye concentration (mg/L), K_L_=Langmuir adsorption constant, K_F_ and n = Freundlich adsorption constant and Freundlich exponent. k_1_ = rate constants for the pseudo-first-order (1/h), k_2_ = rate constants for the pseudo-second-order model (g/mg/h), α = the initial adsorption rate (mg/g/h), β = Elovich constant, q_t_ = the adsorption capacity at time t (mg/g), and C is the intercept.

### Desorption study

A crucial factor in the industrial application of biochar is its ability to sustain multiple adsorption cycles while effectively removing the maximum amount of dye molecules. This is assessed through a desorption study after completing the adsorption process, which undergoes desorption using various pollutants. The regenerated biochar is then reused for subsequent adsorption cycles. Following adsorption, the biochar is referred to as spent biochar. In this study, the desorption efficiency of hydrochloric acid (HCl), sodium hydroxide (NaOH), and deionized water (H₂O) as elutants was evaluated. The procedure involved adding approximately 1 g of spent biochar to a 250 mL Erlenmeyer flask containing 100 mL of the selected elutant. The mixture was stirred at room temperature for one hour, after which the biochar was separated, oven-dried, and prepared for reuse. The remaining solution was analyzed using a UV-vis spectrophotometer to measure the dye concentration, allowing for an assessment of each elutant’s effectiveness in desorbing MB from the biochar.

## Results and discussions

### Biomass characterization

The physicochemical results of ANH with other biomass, such as coconut husk, rice husk, and sunflower husk, were presented in Table 3. The proximate study confirmed that ANH has 4.69 ± 0.30% moisture content, which is lower than other testified biomass such as coconut husk (8.9 ± 1.1)^32^, rice husk (8.09 ± 0.06)^33^ and sunflower husk (7.92 ± 0.24)^34^, respectively. Further, the volatile matter of ANH is observed to be 78.03%, which is higher than that of coconut husk, rice husk, and sunflower husk. The ash content was 5.86%, which is higher than coconut husk and sunflower husk but lower than rice husk. The higher volatile matter and lower ash quantity in biomass suggest that the fuel will ignite easily^35^. Additionally, more volatile biomass suggests that more liquid fuel was produced during pyrolysis. The decreased ash percentage also indicates that there would be less chance of the boiler or furnace becoming clogged^36^. In addition, a larger ash percentage would have transformed into a heat sink and decreased the combustion efficiency. Hence, the lower ash level of ANH improved the fuel’s ability to heat^36^. Additionally, ANH was more suitable as a pyrolysis feedstock since its moisture level is 5.79%, which is lower than the allowed limit (< 10%)^37^. Lastly, the quantity of fixed carbon was found to be 11.42%, which is less than the other recorded biomass ((except rice husk) shown in Table 3. The estimated biomass in Table 3 and the proximate findings from ANH are quite comparable; however, there is a slight discrepancy in the results, which resulted from differences in the biomass component. The elemental results confirmed 49% carbon, 5.57% hydrogen, 0.64% nitrogen, 0.10 sulphur and 44.75% oxygen, respectively. The carbon content is found to be higher than rice husks and sunflower husks but lower than coconut husks. Hydrogen content is also found to be higher than rice husk but lower than sunflower husk and coconut husk. The amount of nitrogen and sulphur content is almost the same range; however, a slight variation is noted due to the chemical composition of biomass. The oxygen content is found to be higher than the biomass testified biomass in Table 3. The low levels of nitrogen and sulphur in the mixture prevented the production of SO_x_ and NO_x_ during pyrolysis^38^. There is a clear relationship between the carbon content and the heating value of fuel because the elemental components of biomass directly affect the heating value^38^. The creation of CO, CO_2_, various pollutants, and particulate matter released into the air, such as volatile organic compounds and nitrogen oxides, is responsible for the oxygen content being slightly greater than the biomass stated in Table 3^39^. It was discovered that the molar ratio of O/C and H/C was found to be 1.49 and 1.21, respectively, which indicates that the emergence of water or oxygenated substances would be higher after pyrolysis^40^. Furthermore, the higher heating value (HHV) of ANH is found to be 18.40 MJ kg^− 1^, which is higher than rice husk (17.26 MJ kg^− 1^) and coconut husk (17.36 MJ kg^− 1^) but lower than sunflower (20.16 MJ kg^− 1^). The higher HHV of ANH establishes maximum heat realise during pyrolysis. The bulk density of ANH is found to be 280 kg m^− 3^, which is higher than that of rice husk and coconut husk but lower than that of sunflower. The higher bulk density of biomass conformed transportation and storage of the sample would be easier. The extractive content stud of ANH confirmed 12.56%, which is lower than rice husk and coconut husk but higher than sunflower husk. Finally, ANH has a higher biochemical percentage than rice husks but lower than coconut and sunflower husks. The larger quantities of cellulose and hemicellulose in ANH suggested that the pyrolysis process produced the most liquid fuel possible^41^.

**Table 3:** Characterisation of ANH and comparison with other reported biomass.

Analysis	Areca nut husk	Coconut husk ^32^	Rice husk ^33^	Sunflower husk ^34^
Proximate analysis (wt%) on a dry basis				
Moisture content	4.69 ± 0.3	8.9 ± 1.1	8.09 ± 0.06	7.92 ± 0.24
Ash content	5.86 ± 0.2	4.6 ± 0.8	34.60 ± 0.06	2.96 ± 0.09
Volatile matter	78.03 ± 0.3	67.8 ± 1.5	52 ± 2	73.2 ± 1.41
Fixed carbon	11.42 ± 0.1	18.7 ± 1.3	5.27 ± 1.02	18.09 ± 0.2
Ultimate analysis (wt% ) on a dry basis				
Carbon	49	55.08	30.4	43.87
Hydrogen	5.57	7.98	4.0	6.29
Nitrogen	0.69	0.22	0.9	1.57
Sulphur	0.10	0.4	0.2	0.1
Oxygen	44.74	36.32	21.1	40.5
O/C	1.49	2.02	1.92	1.44
H/C	1.21	1.13	1.58	1.13
Heating value (MJ kg^−1^)	18.40 ± 0.6	17.36 ± 0.16	17.26 ± 0.01	20.16 ± 0.24
Bulk density (kg m^−3^)	280 ± 10	448.07 ± 24.80	105.5 ± 13.2	838 ± 12.4
Chemical analysis (wt%)	80.57	86	68.95	98.76
Hemicellulose	15.42	53.29	33.07	53.12
Cellulose	53.92	10.98	28.6	24.97
Lignin	11.23	21.73	7.28	20.67
Extractive analysis (wt%)				
Hexane	5.35	9.09	10.8	2.73
Ethanol	7.21	9.91	12.2	6.54
Crystallinity index (%)	47.50	–	–	–

### Thermal stability

**Fig. 3:**
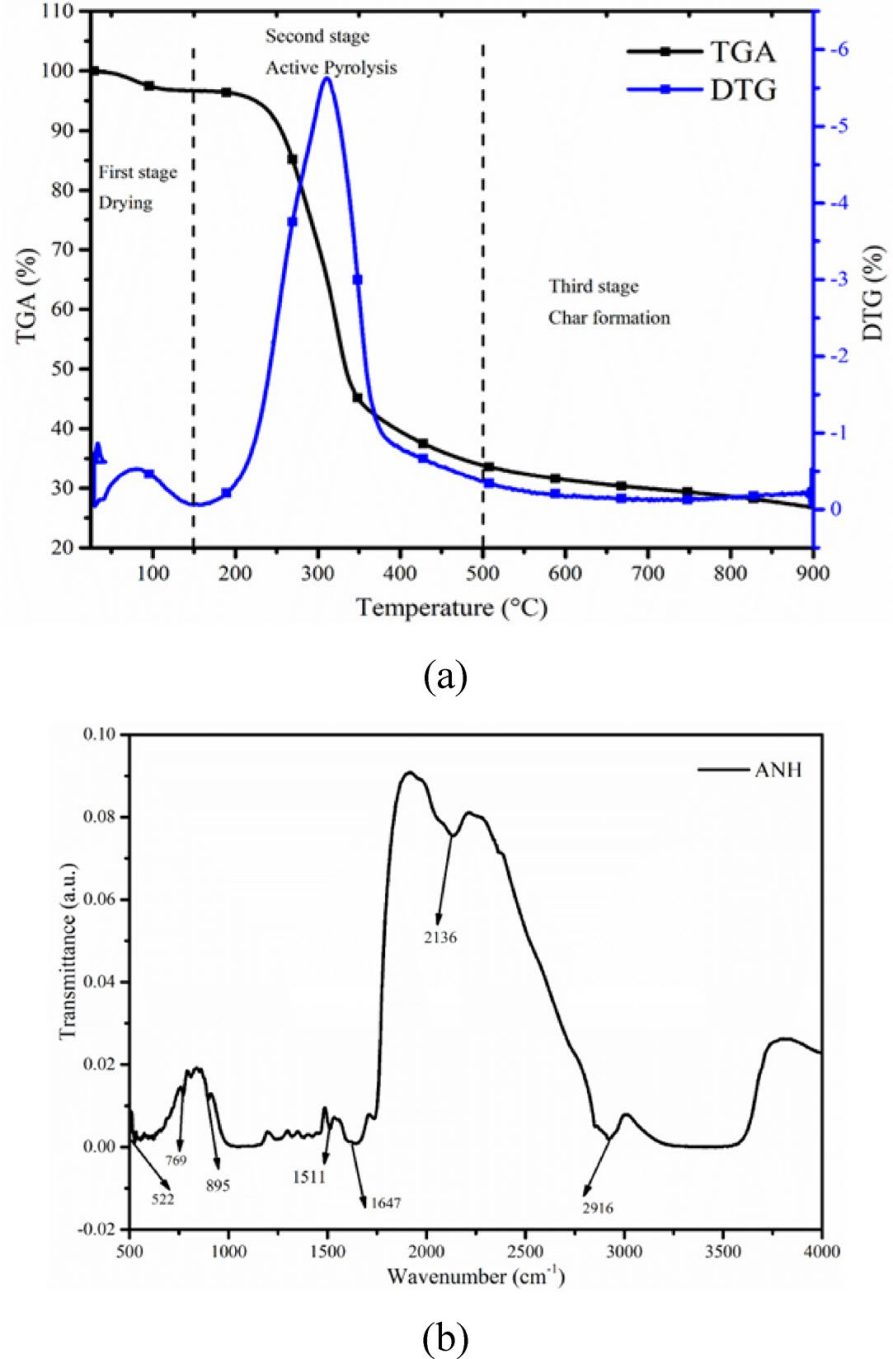
(**a**) TGA profile of Areca nut husk at a heating rate of 10 ℃ min^−1^, and (**b**) FTIR profile of Areca nut husk (ANH).

The thermal breakdown profile of ANH is presented in Fig. 3**(a).** The TGA profile of ANH confirmed that decomposition occurs in three stages, mainly the drying stage (upto 150 ^o^C), active pyrolytic stage (150–500 ^o^C), and finally, passive pyrolytic stage (> 500 ^o^C). At the initial stage, moisture and light volatile matters (lower molecular weight compounds) vaporised upto 150 ℃. Additionally, the TGA profile verified that most components degraded between 150 and 500 °C. Higher molecular weight items, like heavy cellulose and hemicellulose molecules, were separated into low molecular fractions in the second stage (150–500 °C), which resulted in the production of hot volatiles^42^. Additionally, hemicellulose decomposed in the first phase (typically 180–300 °C) and cellulose in the second phase (typically 300–480 °C) as a result of the second stage’s division into lower temperature and moderate temperature zones. Cellulose and lignin are more thermally stable than hemicellulose. At temperatures higher than 500 °C, the hydroxyl phenolic groups that made lignin more thermally stable might disintegrate and produce charcoal. The first peak was seen as a result of the elimination of moisture and extremely light volatile chemicals up to 150 °C (DTG analysis of ANH) and displayed in Fig. 3**(a).** The temperature range of 250–500 °C was found to be the peak for cellulose and hemicellulose breakdown. Lastly, because of its better thermal stability, lignin degrades at temperatures higher than 500 °C. The lignin fragmentation is attributed to the breaking of C-O bonds, which results in the formation of substances with one oxygen atom^43^. Finally, the side chain C-C bonds between the carbon atom and aromatic ring are broken at 327–380 ^o^C by the methoxy cleavage, which contributes molecules with two oxygen atoms^43^. In moderate reaction conditions, the degradation of comparatively weaker linkages (alkyl-aryl ether) leads to the creation of biochar. Lignin contributes more aromatic compounds, short organic molecule chains, and gaseous products, including CO and CH_4_, after thermal depolymerization. Sharp peaks cannot occur because of the lesser lignin content compared to hemicellulose and cellulose and because of lignin’s slower rate of breakdown^43,44^. TGA and DTG profiles confirmed 6.22, 77.12 and 16.66% decomposition in the first, second and third stages, respectively. The results obtained from the TGA also confirmed the maximum decomposition of biomass in the second stage; however, moisture content, volatile matter, and fixed carbon listed in Table 3 have a good match with the TGA results.

### FTIR analysis

The presence of phenols, water, ester, ether, acids, alkanes, aliphatic chemicals, and aromatic products was verified by FTIR analysis of ANH **(**Fig. 3**(b)).** The -OH deformation-assigned adsorption band 3000–3634 cm^− 1^ confirmed the presence of water, acids, phenols, and aromatic chemicals^42^. Alkane was established by the peak between 2850 and 3000 cm^− 1^, attributed to C-H stretching, whereas the carbonyl/carboxylic acid continuity was demonstrated by the peak between 1750 and 11,647 cm^− 1^, attributed to C = O stretching^45^. The presence of aromatics and alkene was confirmed by the adsorption band 1511 cm^− 1^ attributed to the C = C stretching vibration, whilst the presence of alkyne was indicated by peaks between 1455 and 1230 cm^− 1^ attributed to the C$\:\equiv\:$C deformation vibration^35^. Esters and ether were identified by the adsorption band 895 cm^−1^, which was attributed to C = O stretching and deformation vibration. In contrast, the survival of mono and polycyclic substituted aromatic compounds was indicated by peaks 769 and 522 cm^− 1^, which were attributed to O-H bending^45^.

### Biochar characterization

The yield and physicochemical properties of biochar are listed in Table 4. The yield of biochar was found to be 34.12 ± 0.46 wt% and 18.24 ± 0.12 wt% at 600 and 800 ^o^C, respectively. The yield of biochar at 600 ^o^C was found to be higher than 800 ^o^C due to possibly incomplete pyrolysis of biomass^46^. At lower temperatures (600 ^o^C), certain reactions that produce desired products, such as hydrogen, may be favoured over reactions that lead to the formation of undesired by-products. however, at higher temperatures (800 ^o^C), secondary reactions like cracking and further decomposition may become more prevalent. These secondary reactions can lead to the formation of smaller molecules, potentially reducing the yield of specific target products^47^. Further, the moisture content of biochar derived at 600 ^o^C (ANHB600) was found to be higher (5.11%) than biochar derived at 800 ^o^C (ANHB800) (4.47%). At lower temperatures, the moisture within the biochar may not evaporate as effectively. This can lead to condensation of water vapour on the biochar particles, causing an increase in moisture content^48^. Further, it was noticed that the moisture content of acid-treated biochar at 600 ^o^C (ANHBA600) has lower moisture content (9.60%) than the acid-treated biochar at 800 ^o^C (ANHBA800) (12.52%) due to possibly increased surface area, which absorbs the moisture^48^. The ash content of ANHB600 was found to be higher than the ANHB800 due to partial pyrolysis of biomass^49^. However, the ash content in ANHBA600 was found to be higher (9.96%) than in ANHB800 (10.73%). The acid dissolves the mineral components present in the biochar, which are typically responsible for the ash content. These minerals can include calcium, magnesium, potassium, sodium, and various metal oxides. The acid converts these minerals into soluble ions^50^. The elemental analysis of biochar showed an improved carbon content and reduced oxygen content against temperature. It was found that by increasing temperature from 600 to 800 ^o^C, around 4% carbon content increased, and around 3.13% oxygen content was found to be reduced due to the elimination of impurities present in biochar found at 600 ^o^C. The variations in carbon and oxygen content observed due to mainly enhanced volatilization of oxygen-containing compounds, including water and organic acids, are more pronounced^51,52^. This removal of volatile components contributes to a decrease in the oxygen content of the resulting biochar. Moreover, the promotion of dehydration reactions at higher temperatures results in the expulsion of water from the biomass, further reducing the oxygen content^52^. Biochar produced at higher temperatures exhibits greater stability and is less prone to further oxidation, thus contributing to lower oxygen content^51^. A similar trend and phenomenon was noticed for acid-treated biochar. Acid-treated biochar showed higher carbon content due to the removal of ash content without treated biochar; however, 800 ^o^C stands as the best biochar for further application. HHV of biochar increased with an increased pyrolysis temperature due to the increased elemental composition of biochar (Table 4). Biochar produced at 600 and 800 ^o^C was found to have HHV values of 32.62 and 33.02 MJ kg^− 1^, respectively. The biochar’s heating value tends to rise at higher pyrolysis temperatures because of the more intense carbonisation process^53^. Organic biomass is subjected to thermal breakdown in a low-oxygen environment during pyrolysis. A more concentrated and energy-dense carbon structure is left behind at higher temperatures when more volatile elements, such as water and organic gases, are forced out^54^. A larger carbon content in biochar is produced by the enhanced elimination of volatile materials, which increases the heating value^54^. Essentially, a higher temperature during pyrolysis increases the effectiveness of carbonisation, resulting in a biochar product with a larger potential for energy release after fuel usage^53^. A similar trend and behaviour was noticed in the case of acid-treated biochar. ANHBA800 has a higher HHV (34.30 MJ kg^− 1^) than the ANHBA600 (33.14 MJ kg^− 1^) due to the effectiveness of carbonisation. The bulk density of biochar was also found to be increased with an increased pyrolysis temperature. The biochar produced at 600 and 800 ^o^C was found to have bulk densities of 111.17 kg m^− 3^ and 138.93 kg m^− 3^, respectively. Higher pyrolysis temperatures tend to increase the bulk density of biochar, mainly due to the decrease of volatile matter and the rise in carbon content during the pyrolysis process^55^. A denser carbon structure remains in the biochar as a result of the more volatile substances, such as water and organic gases, being pushed away by the rising temperature^55^. The elimination of these vaporous materials causes the biochar’s total volume to decrease, which raises the bulk density. A similar trend and behaviour was noticed in the case of acid-treated biochar. ANHBA800 has 97.49 kg m^− 3^ than ANHBA600 (85.49 kg m^− 3^). The bulk density of acid-treated biochar was found to be reduced to that of raw biochar due to the elimination of various unwanted impurities (mineral content, ash content, etc.). The surface area of biochar was found to be increased with an increase in pyrolysis temperature and acid concentration. The biochar produced at 600 and 800 ^o^C was found to have a BET surface area of 99 and 112 m^2^ g^− 1^, respectively (Table 4**)**. The surface area of biochar increases with higher pyrolysis temperatures because the process removes volatile components, leading to the development of a more porous and structured carbon matrix^56^. Similarly, the acid-treated biochar at 600 and 800 ^o^C shows an increase in BET surface area (Table 4**)**. The ANHBA800 has 205 m^2^ g^− 1^ BET surface area compared to ANHBA600 (140 m^2^ g^− 1^) (Table 4**)**. The surface area of biochar often increases with acid treatment due to the removal of ash and mineral impurities, resulting in a more porous structure^57^. Acid treatment can dissolve minerals and ash content present in the biochar, expanding pores and exposing more surface area. Additionally, the acid treatment may create new functional groups on the biochar surface, contributing to increased porosity and surface complexity^57^. From the analysis, it was observed that rather than normal biochar, acid-modified biochar showed a more enlarged porous structure and larger surface morphology at 800 ℃ (ANHBA800). The pH is a measure of the concentration of hydrogen ions (proton ions) in the solution^58^. The pH of biochar derived at 600 and 800 ℃ was found to be 10.14 and 10.74, respectively, and listed in Table 4. From the results, it was noticed that the pH of the biochar increases with the increase in temperature from 600 to 800 C, which concludes the alkaline nature of biochar. This is mostly due to the process’s ability to degrade acidic organic molecules found in biomass, eliminate volatile acidic components, and activate alkaline minerals. Consequently, the final biochar product becomes more alkaline and less acidic^59,60^. A similar phenomenon happened in the case of acid-treated biochar. The water-holding capacity (WHC) of biochar depends on the composition, structure and porosity of biochar^61^. The water-holding capacity of biochar increases with an increase in the pyrolysis temperature as the surface area, and the porosity of the biochar increases due to the removal of the functional group (Table 4**).** The biochar obtained at 600 and 800 ^o^C has 25 and 26% WHC due to an increased porosity of biochar which is supported by BET results. In the case of acid-treated biochar, again, the porosity and surface area increased, which resulted in an increased WHC.

**Table 4:** Characterisation of Biochar at temperatures 600 and 800 ℃.

Analysis	ANHB (600 ℃)	ANHB (800 ℃)	ANHBA (600℃)	ANHBA acid (800 ℃)
Yield (wt%)	34.12 ± 0.46	18.24 ± 0.12	–	–
Proximate analysis (wt%) on dry basis				
Moisture content	5.11 ± 0.25	4.47 ± 0.08	9.60 ± 0.42	12.52 ± 0.46
Ash content	27.23 ± 2.32	22.25 ± 0.52	9.96 ± 0.45	10.73 ± 0.26
Volatile matter	25.01 ± 0.21	28.04 ± 0.43	20.11 ± 1.3	17.07 ± 0.21
Fixed carbon	42.65	45.24	59.61	60.71
Ultimate analysis (wt% ) on dry basis				
Carbon	59.12	62.93	67.15	68.66
Hydrogen	1.542	1.474	2.039	1.810
Nitrogen	1.213	1.21	1.182	1.293
Sulphur	–	–	–	–
Oxygen	38.31	35.18	29.11	28.73
High Heating Value (MJ kg^− 1^)	32.62 ± 1.1	33.02 ± 0.98	33.14 ± 1.2	34.30 ± 1.56
Bulk Density (kg m^− 3^)	111.17 ± 0.547	138.935 ± 7.508	85.495 ± 3.018	97.90 ± 3.373
BET Surface Area S_BET_ (m^2^/g)	99.10	112	140	205
pH	10.14	10.74	6.46	6.67
Water holding capacity	25.4	26.9	26.12	26.98

### Thermal stability of Biochar

**Fig. 4:**
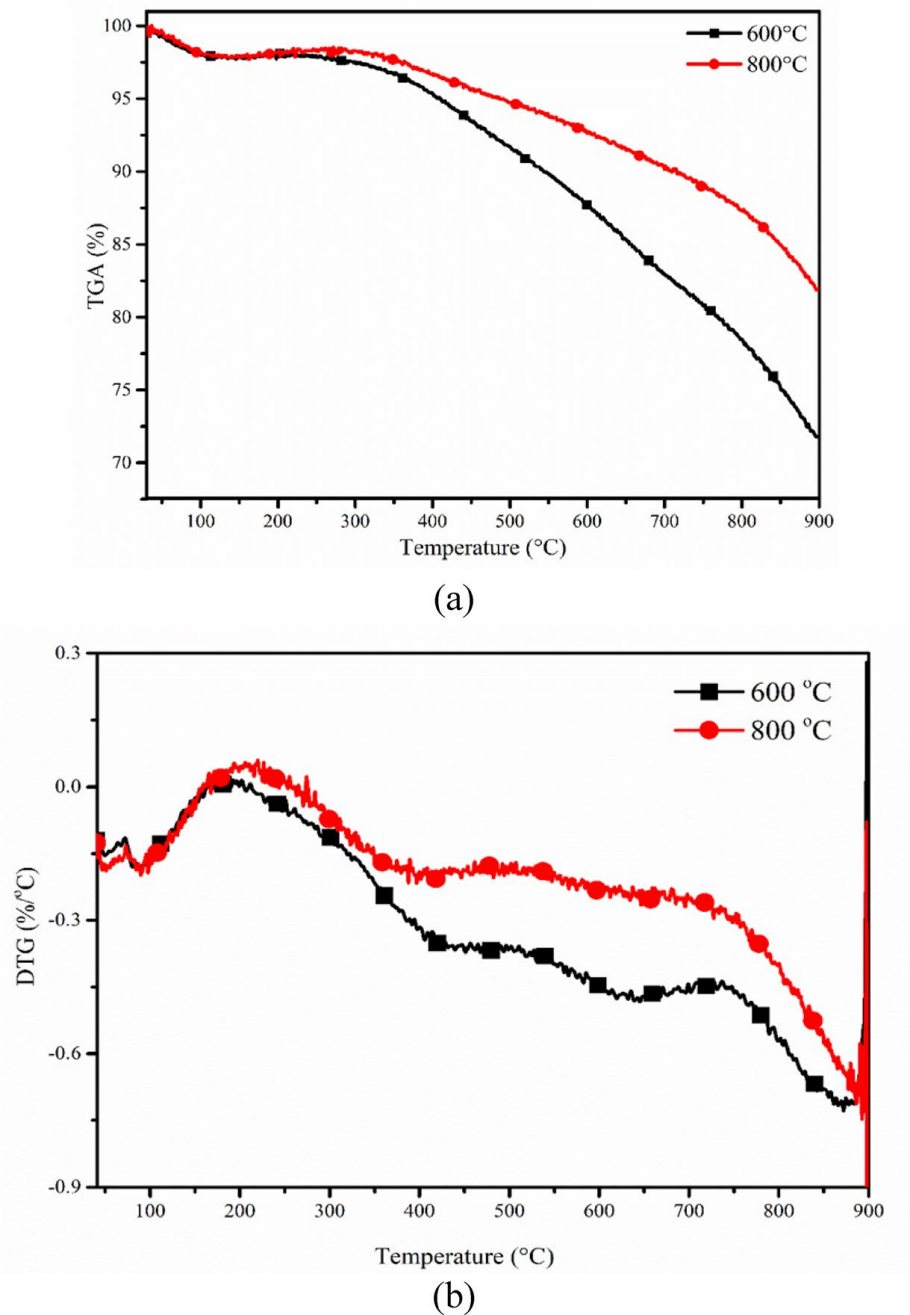
(**a**) TG profile of ANHB and (**b**) DTG profile of ANHB at 600 and 800℃.

The thermal stability profile of ANHB at 600 and 800 ℃ is presented in Fig. 4**(a) and (b)**. The thermal stability profile of ANHB displayed two-stage decomposition known as drying (first stage) and decaying (second stage). Further, the first stage (30–150 ℃) showed the elimination of humidity from the biochar. However, the second stage (150–900 ℃) confirmed the decomposition of biochar against temperature. This phenomenon is observed due to the elimination of impurities (volatiles or non-combustible matter) present in biochar^62^. From the graph (Fig. 4**(a) and (b))**, it was observed that the biochar obtained at a higher temperature (800℃) has higher thermal stability due to complete pyrolysis (removal of oxygen, hydrogen and volatiles) than 600℃. However, the biochar obtained at a lower temperature (600 ℃) comprises impurities in the form of volatiles that reduce the thermal stability of biochar. In the first stage, ~ 1.36 and 1.88% decomposition were recorded at 600 and 800 ℃, respectively, due to the elimination of primary hydroxyl groups or moisture within the temperature range of 30–150 ℃. The overall decomposition was about 30% and 15% for biochar produced at 600℃ and 800℃, respectively, attributed to impurity removal. Biochar made at lower temperatures retained certain impurities, such as hydrocarbons, due to incomplete pyrolysis caused by limited heat and mass transfer, leading to higher decomposition. DTG thermograph revealed that significant biochar decomposition occurred between 100–350℃, with peak decomposition at 175℃ for 600℃ biochar and 209℃ for 800℃ biochar. Mishra et al. (2022) studied the characterisation of biocarbon derived from chicken feather oil and spent coffee ground oil-derived biochar, which displayed a similar trend^62^.

### FTIR study

**Fig. 5:**
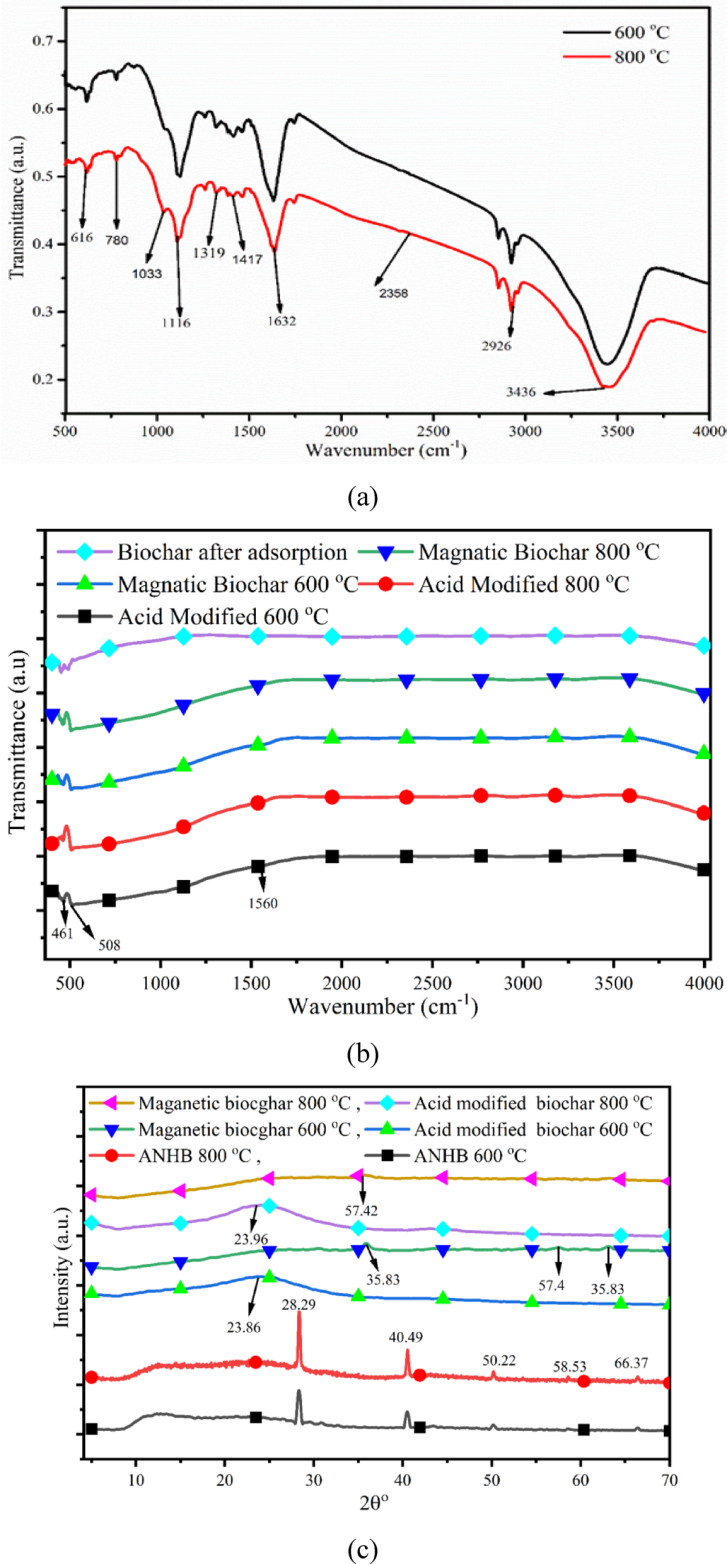
(**a**) FTIR analysis of ANHB at 600 and 800 °C, (**b**) FTIR analysis of acid-treated, magnetic biochar and biochar after adsorption, and (**c**) XRD analysis of raw, acid-modified and magnetic biochar at 600 and 800 °C.

FTIR spectra of biochar prepared at 600 and 800 ℃ were shown in Fig. 5**(a).** It was found that the functional group present in the biochar substantially altered concerning pyrolysis temperature. The peak found at 3436–3695 cm^− 1^ was linked with O-H stretching, establishing the presence of moisture, acids and phenolic constituents^62^. Further, a peak was observed at 2926 cm^− 1^, indicating C-H stretching, suggesting the presence of cellulose and hemicellulose. Also -COO^−^ anti-symmetric stretching of amino acids and alkanes was demonstrated at a peak of 1632 cm^− 1^, indicating pyrolysis of biomass, which decomposed hemicellulosic matter and removed carboxylic acids while increasing the temperature^6,63^. A peak ranging from 1323 to 1390 cm^− 1^ indicated -O-C-O- stretching vibrations, which decreased with an increase in temperature during pyrolysis, suggesting loss due to ignition and carbon condensation^64^.

Peaks corresponding to in-plane bending of carbonyl and carbonate were identified at 1417 cm⁻¹ and 1116 cm⁻¹^63^. The C-O bond of alcohol and ether groups at 800 cm⁻¹, along with the pyridine group of heterocyclic nitrogen compounds at 780 cm⁻¹, remained intact at higher temperatures. A peak at 2358 cm⁻¹ was linked to the asymmetric stretching of CO₂^64^,, while aromatic C-C stretching at 1507 cm⁻¹ and C-Cl bending at 581 cm⁻¹ disappeared at lower temperatures but re-emerged at 800℃^65^. Shafiq et al. (2021) investigated the influence of varying pyrolysis temperatures on the properties of *Parthenium hysterophorus-*derived products and reported a comparable trend in FTIR analysis^65^. Similarly, Chatterjee et al. (2020) studied the effect of pyrolysis temperature on acoustic-based amination of biochar, and similar results were obtained^66^. The acid-treated and magnetic biochar derived at 600 and 800 ^o^C is listed in Fig. 5**(b**). Acid-treated and magnetic biochar has 461, 508, 1560, 2932 and 3302 cm^− 1^ peaks, which confirms the presence of poly and mono aromatic compounds, aromatic C-C stretching, C-H stretching and -OH functional groups, respectively. The variations in transmittance for biochar samples subjected to magnetic and acid modifications at 600 °C and 800 °C. Magnetically modified biochar exhibits higher transmittance across all wavenumbers compared to acid-modified biochar, indicating differences in surface chemistry and functional groups^67^. Further, at 800 °C, magnetically and acid-treated biochar show reduced oxygen-containing functional groups due to thermal degradation, but the acid-modified biochar exhibits lower transmittance, suggesting more extensive structural breakdown. The spectra also reveal that magnetic modification helps retain surface functionalities better than acid treatment, which aggressively removes functional groups, reducing transmittance intensity. Higher pyrolysis temperatures lead to a more carbonised structure, diminishing characteristic peaks. The shift in transmittance patterns between 600 °C and 800 °C indicates temperature-dependent chemical transformations^13^. Additionally, the broader spectral differences in acid-modified biochar suggest enhanced porosity and structural changes, whereas magnetic biochar maintains a more stable framework with preserved functional groups at elevated temperatures^67^. Overall, biochar has many functional groups which are used in a variety of applications. However, acid-treated biochar helps reduce ash content, oxygen functional groups and other ketonic groups. The magnetic biochar exhibits higher transmittance across all wavenumbers, indicating differences in surface chemistry and functional groups^13^. The FTIR analysis of biochar obtained after adsorption confirmed almost the same functional group except for the intensity. Raw, acid-treated and magnetic biochar has a higher depth of intensity over biochar derived after adsorption text confirmed blockage of the posre of the biochar, which further limits the adsorption potential of biochar.

### XRD analysis

XRD study demonstrates the structure of raw, acid-treated and mangetic biochar at 600 and 800 ^o^C which is displayed in Fig. 5**(c).** The peak is observed using Match software (Crystal Impact, Germany) (version version 4.1 (Build 309). For the XRD analysis, biochar manufacture via slow pyrolysis of ANH at 600 and 800 ℃ with 45 min holding time is carried out. A range of mineral crystals and various inorganic components were found in the XRD study. Fluorite, graphite and chlorapatite were obtained at the peak 28.33 and 28.39 ^o^ for 600 and 800℃, respectively^68^. The second peak was observed at around 40.51 and 40.64 ^o,^ indicating the presence of mineral gibbsite. Similar peaks were found at 50.22 and 50.25^o,^ representing the presence of calcite, bayerite and hydrobiotite-related components. Further peaks corresponding to apophyllite, pyrophyllite and barite were obtained at 58.56 and 58.65^o,^ respectively^68^. The various peaks, along with their intensities, were decreased along with a marginally more prominent background and signal-to-noise ratio. The change in peaks depicts how minerals degrade at high temperatures and produce new elements. Between 600 and 800 ℃, materials get degraded, and the peak in the XRD spectra biochar at the respective temperatures is obtained as the material is crystalline^68^. XRD data shows different mineral elements, including iron, sulphur, calcium, magnesium, sodium, silicon, and carbon. It was found that temperature has a higher influence on the structural constituent of biochar^68^. Armynah et al. (2018) did a physicochemical analysis of biochar obtained from rice husk at different temperatures and different heating rates where similar trends were observed^69^. Shang et al.(2016) investigated magnetic biochar derived from herb residue, and various peaks were found at different temperatures, and a similar XRD analysis was observed^70^. Acid-modified biochars at temperatures retain their largely amorphous nature, as evidenced by their broad humps and lack of sharp peaks. However, the 800 °C sample shows a slightly more ordered structure, likely due to thermal reorganisation facilitated by acid treatment^70^. Further, magnetic biochars also present mostly amorphous patterns; the sample prepared at 800 °C shows a relatively flat baseline with no significant peaks, while the 600 °C sample shows a mildly elevated background, which could be attributed to the partial crystallization or improved dispersion of iron species in the carbon matrix. However, no distinct crystalline iron oxide peaks are observed, indicating that the magnetic phase may exist in nano-sized^70^. Overall, the increase in pyrolysis temperature enhances the crystallinity of the biochars, particularly for the unmodified sample, while acid and magnetic modifications maintain a largely disordered carbon structure^68^.

### SEM analysis

**Fig. 6:**
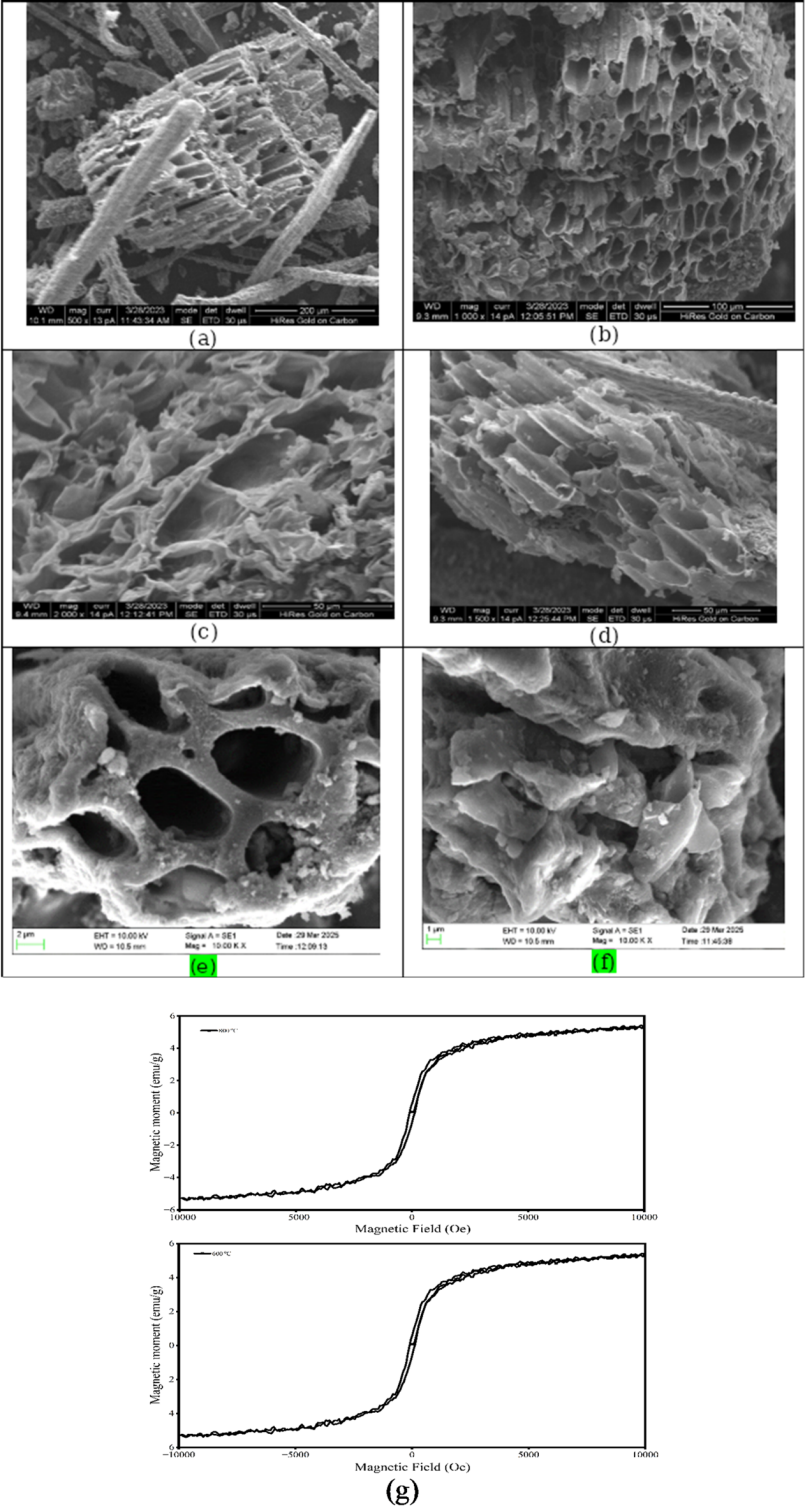
SEM analysis of ANHB (**a**) 600 ℃ (**b**) 800 ℃ (**c**) acid modified 600 ℃ (**d**) acid modified 800 ℃, (**e**) Magnetic biochar at 600 °C and (**f**) magnetic biochar at 800 °C. (**g**). The magnetic moment of magnetic biochar derived at 600 and 800 °C at 30 °C.

The SEM images exhibit distinct morphological differences, highlighting variations in porosity, structure, and surface texture. Figure 6**(a**,** b**,** c and d)** demonstrates the structure of raw and acid-modified biochar produced at 600 and 800 ℃. Figure 6**(a)** reveals a fibrous and entangled network, indicative of a cellulose-based structure or partially carbonised biomass. In contrast, Fig. 6 (b) presents a highly porous architecture, suggesting an activated carbon or biochar material with an enhanced surface area, ideal for adsorption. It was observed that the uniformity of the biochar and the rate of aromatization improve with the pyrolysis temperature because of the volatilisation of oxygen and hydrogen from the lignocellulosic components with lesser aromatisation and lignin, leading the remnant carbon to generate new aromatic bonds^71^. With the rise in temperature, the uneven fold structure changes to the regular layer of the micro-particle and smaller pores in the biochar^72^. The configuration of biochar changes during pyrolysis due to many reactions, such as dehydration, decarboxylation, and decarbonylation^73^. The biochar formed at 800 ℃ has a higher porous surface due to the vaporization of organic material^74^. The pores were generated and alerted due to the combustion of the organic material during pyrolysis. Biochar has a higher porosity than the Areca nut husk biomass. By elevating the pyrolysis temperature from 600 to 800 ℃, the overall surface configuration was changed, and flake-like surfaces were formed. The biochar produced at 600 and 800 ℃ has an uneven surface morphology^75^. The process where micropores are adhered with condensed volatile and decompose materials, which to a certain extent block the pores, is the reason for the flaky surface of biochar produced at elevated temperatures. Figure 6**(c) and (d)** display fractured, layered structures, likely representing pyrolyzed biomass with preserved microstructures that influence adsorption and catalytic performance. It was observed that the breaking of chemical bonds during pyrolysis modified the structure and pores of the biochar^76^. Acid treatment exhibited maximum effect due to the additional release of water vapour through the dehydration of sulphuric acid, and pore wall destruction was observed. As a result, the pore size also enlarged in acid-modified biochar. Acid treatment increases the surface morphology, enlarges the porous structure, and improves the surface of biochar with oxygenated functional groups such as carboxylic, hydroxyl, carbonyl, etc., which results in the generation of negative surface charge of the biochar, and this leads to an increased rate of adsorption of the cationic species^77^. Modification with acids produces positive sites on the exterior surface of biochar, and protonation of surface hydroxyl groups enhances the adsorption capacity for cadmium and lead^78^. Biochar obtained at 800 ℃ showed more enlarged pores after acid modification^79^. Suri et al. (2014) reported that biochar derived from oil palm, despite having a lower surface area, demonstrated a higher adsorption capacity for heavy metals compared to rice husk biochar^80^ Peiris et al. (2019) studied the effect of acid alteration on the physical and chemical properties of biochar obtained from tea waste produced through slow pyrolysis and similar results were obtained^81^. Figure 6**(e and f)** showed the surface morphology and characteristics of the magnetic biochars. Compared with Fig. 6 (a-d), Fig. 6(e and f) showed bright spots, which gives the idea that some degree of conductivity exists in biochar. The surface morphology of ANHB600 and ANHB800 exhibit macro porosity. During iron oxide formation, some biochar pores become blocked by iron oxide particles that form in the biochar^67^. Mohan et al. (2014) also stated the same result for the magnetization of biochar derived from oak wood and oak bark^13^. These variations in morphology directly impact the material potential, such as adsorption, catalysis, or energy storage, with differences in pore size and surface properties playing a crucial role in their performance and efficiency.

### Acid modification and magnetic properties of Biochar

Ash content is a measure of the mineral content and other inorganic impurities present in the biochar, which is estimated using a programmable muffle furnace. The characterisation result of biochar, shown in Table 5, revealed that the ash content is higher, reducing biochar efficiency and increasing the risk of clogging. The acid modification of biochar produced at 600 and 800 ^o^C was carried out at different concentrations (1, 5, 7 and 9 M H_2_SO_4_ ) to reduce the ash content (Table 5). The acid modification provides more oxygen-enriched functional groups (carboxylic groups) on the surface of biochar and enhances biochar ability^82^. From Table 5, it was found that at 5M H_2_SO_4_ showed promising ash content (9.45 and 10.30 wt% for 600 and 800 ^o^C) and increased BET surface area (140 and 265 m^2^ g^− 1^ for 600 and 800 ^o^C) compared to other concentrations. The acid treatment typically involves immersing the biochar in acidic solutions, which removes ash and mineral impurities, leading to a cleaner and more porous biochar structure^56^. Higher ash content in adsorbents can reduce methylene blue (MB) dye adsorption by blocking active sites and lowering surface area. However, specific mineral components in ash may enhance adsorption through electrostatic interactions. The overall effect depends on ash composition, porosity, and functional groups influencing dye binding efficiency in aqueous solutions. The BET surface of magnetic biochar derived at 800 ^o^C was found to be 385 m^2^ g^− 1^. Magnetic biochar exhibits increased surface area primarily due to the incorporation of iron-based nanoparticles, which act as templates or catalysts during pyrolysis. These particles inhibit the collapse of pore structures and promote the formation of mesopores and micropores. Additionally, the presence of metal oxides facilitates the release of volatile matter, further enhancing pore development^83^. The magnetic modification also prevents the agglomeration of biochar particles, maintaining a more dispersed and porous structure. As a result, the biochar surface becomes more accessible, improving its surface area and making it more effective for applications such as adsorption and catalysis^83^. Further, the BET surface area of biochar derived at 800 ^o^C was found to be 340.60 m^2^ g^− 1^ after adsorption. The BET surface area decreases after adsorption because the adsorbate molecules occupy the available pores and active sites on the biochar surface. This pore blockage reduces the accessible surface area for nitrogen adsorption during BET analysis, indicating successful adsorption and reduced porosity due to the presence of retained adsorbate molecules^83^.

The applicability of ferromagnetic materials is determined by the features of their hysteresis loops derived from magnetization (M) versus applied magnetic field (H) plots. Magnetisation measurements were conducted at 30 °C using a Vibrating Sample Magnetometer (VSM). From Fig. 6(g), the saturation magnetization values for magnetic biochar at 600 and 800 ^o^C were found to be 45.20 and 5.39 emu/g, respectively. Compared to 600 ^o^C biochar, 800 ^o^C biochar showed higher saturation magnetization, probably due to a larger percentage of iron oxides in its composite structure. Mohan et al. (2014) showed that the magnetic biochars derived from oak wood (MOWBC) and oak bark (MOBBC) exhibited distinct magnetic properties, as measured by the use of vibrating sample magnetometry (VSM). MOWBC showed a higher saturation magnetization of 8.87 emu/g compared to 4.47 emu/g for MOBBC, indicating greater magnetic responsiveness due to a higher iron oxide content^13^. Further, Chen et al. (2011) reported that the magnetic biochar performed 91.30% phosphate adsorption and 94.60% elimination of organic contaminants (such as methylene blue) from aqueous solutions. After five regeneration cycles, it maintained over 85% efficiency and allowed for quick separation, demonstrating exceptional reusability with a surface area of 326.5 m²/g and saturation magnetization of 18.2 emu/g^84^.

**Table 5:** Acid modification of Biochar at different concentrations of acid.

Molarity (M)	Ash content (600 ℃) (wt%)	BET surface area (m^2^/g)	Ash content (800 ℃) (wt%)	BET surface area (m^2^/g)	Magnetic biochar BET surface area (m^2^/g)	BET surface area (m^2^/g) after adsorption
1M H_2_SO_4_	8.80 ± 1.10	–	12.80 ± 1.21	–	–	–
5 M H_2_SO_4_	9.45 ± 1.10	140	10.30 ± 1.10	265	385	340.60
7 M H_2_SO_4_	9.96 ± 1.20	–	12.2 ± 1.10	–	–	–
9 M H_2_SO_4_	9.5 ± 1.20	–	11.07 ± 1.20	–	–	

### Batch adsorption studies

#### Influence of dosage of Biochar on methylene blue dye removal

**Fig. 7:**
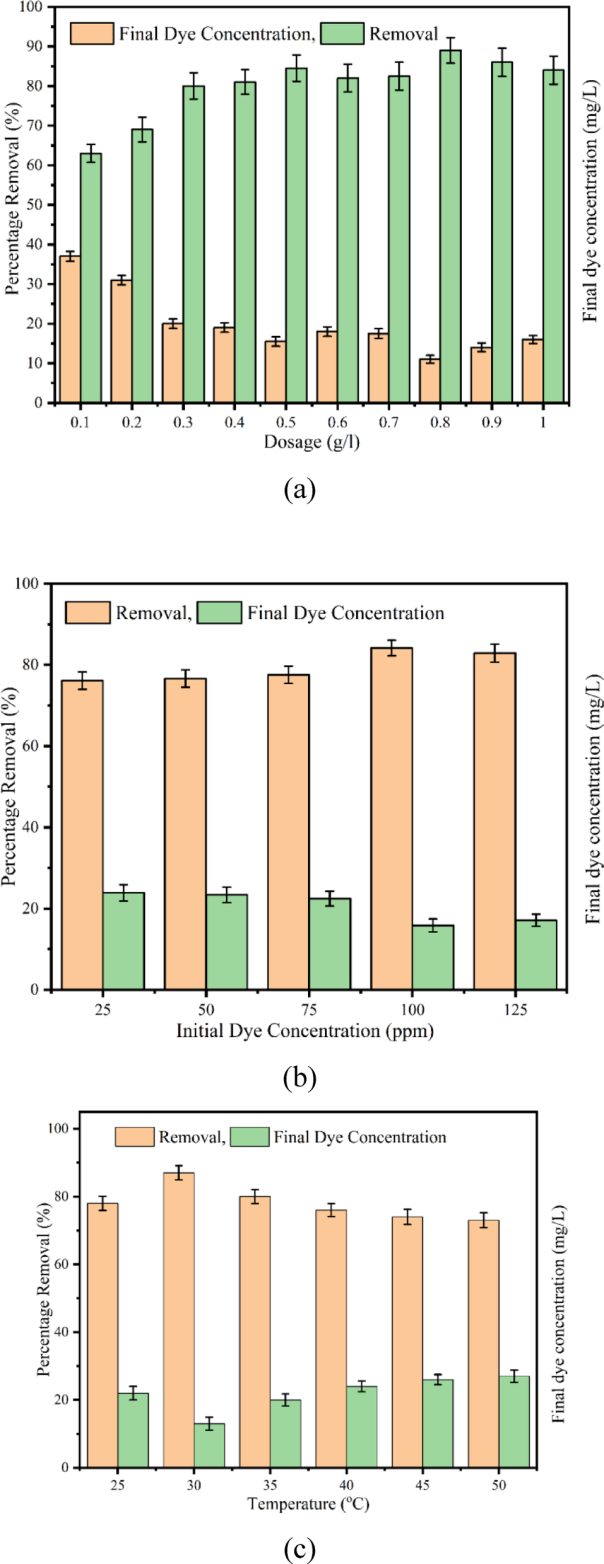
(**a**) Effect of biochar dosage at constant pH of 7, concentration and temp At pH 7, t = 60 min, C_0_ = 100 ppm, T = 30 °C. (**b**) Effect of initial dye concentration at constant pH = 7, 0.3 g/L, t = 60 min and T = 30 °C. (**c**) Effect of temperature at pH 7, t = 60 min, C_0_ = 100 ppm, T = 30 °C.

The effect of biochar dosage on the exclusion of MB dye was evaluated by varying the biochar dosage from 0.1 to 1 g/L. To minimise complexities arising from acidity or alkalinity, the solution was maintained at a neutral pH. Prior to the experiment, the dye concentration was set to 100 ppm. The test was conducted under normal daylight conditions at a fixed temperature of 30 °C (room temperature) for one hour and illustrated in Fig. 7**(a).** It was noticed that the adsorption efficiency increased with higher biochar dosages due to the increased available adsorption sites and enhanced surface area. At a dosage of 8 g/L, the biochar achieved 89% removal efficiency, but further increases led to a decline. Although the highest efficiency was observed at 8 g/L, the remaining experiments were conducted using a dosage of 3 g/L. This decision was based on prioritising efficiency and scalability over optimal performance to align with the study’s focus on commercial applicability. From Fig. 7**(a)**, it is evident that the increase in removal efficiency between 2 and 3 g/L was more significant than any other range, justifying the selection of 3 g/L for subsequent experiments (80% adsorption capacity). Similar findings were reported on the impact of adsorbent dosage on MB dye removal using wet-torrefied microalgal biochar. They determined that a dosage of 1 g/L achieved an 85.47% removal efficiency for MB dye^85^. Thillainayagam et al. (2023) explored the adsorption of methylene blue dyes using chlorella vulgaris microalgal biochar and reported a maximum efficiency of 95.80% at 9 g/l dosage. However, they considered 3 g/l as the optimum for further study^21^.

#### Influence of initial dye concentration on methylene blue dye removal

The effect of the initial methylene blue dye concentration on its removal efficiency was evaluated by varying the initial dye concentration in the liquid medium. To eliminate complications related to pH, the medium was maintained at a neutral pH. As previously determined, the biochar dosage was fixed at 0.3 g/L, and the experiment was conducted at an optimal temperature of 30 °C. The results obtained after a one-hour experiment are presented in Fig. 7**(b).** The plot indicates a gradual increase in removal efficiency with higher initial dye concentrations. This improvement can be attributed to the enhanced driving force between the dye molecules and the biochar, as well as the higher concentration gradient, which facilitated the dye’s passage through the mass transfer barrier, thereby increasing the removal efficiency^86,87^. The maximum removal efficiency, approximately 85.74%, was observed at an initial dye concentration of 100 ppm. In comparison, prior studies reported similar findings in biosorption experiments. Studies reported maximum adsorption capacities of 139.11 mg/g at 2000 ppm for de-oiled algal biomass and 107.50 mg/g for *Sargassum sp.* algae [38, 39], while *Chlamydomonas moewusii* microalgal biomass exhibited 212.41 mg/g for methylene blue removal, consistent with the Langmuir isotherm model and pseudo-third-order kinetics^88^.

#### Influence of temperature on methylene blue dye removal

The influence of temperature on the elimination of MB dye was evaluated at 25 to 50 °C with an increment of 5 °C. The biochar concentration (0.3 g/L) was maintained constant throughout the study. An initial dye concentration of 100 ppm was used, and the pH of the medium was kept neutral to eliminate the effects of acidity or alkalinity. The results shown in Fig. 7**(c)** indicated that the removal efficiency increased from 25 to 30 °C. However, beyond 30 °C, a decline in efficiency was observed. This reduction may be recognised as the breakdown of intermolecular hydrogen bonds at higher temperatures, weakening the interaction between the dye molecules and the biochar surface. Consequently, the highest removal efficiency of 87.10% was achieved at 30 °C, which was determined to be the optimal temperature due to its proximity to room temperature, ease of operation, and reduced energy requirements. In a study on dye removal using dead and living biomass of *Phaeodactylum tricornutum microalgae*, the dead biomass demonstrated better removal efficiencies, with capacities of 18.90, 66.40, and 19.60 mg/g for methylene blue, crystal violet, and safranin, respectively. This was attributed to the difficulty of dye molecules penetrating living biomass, further highlighting the efficiency of non-living adsorbents in dye removal applications^89^.

#### Influence of contact time on methylene blue dye removal

**Fig. 8:**
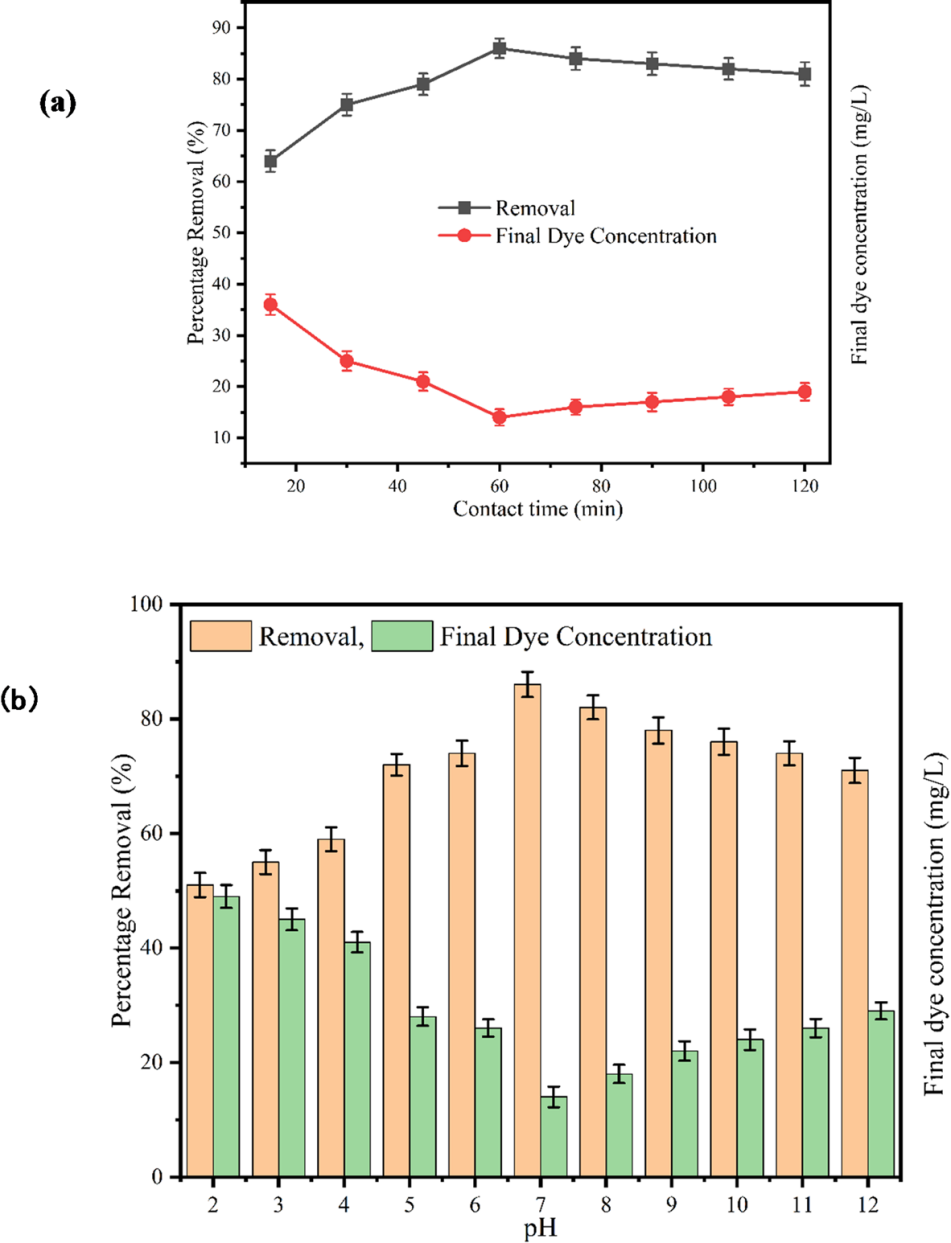
(**a**) Effect of contact time at pH 7, t = 60 min, C_0_ = 100 ppm, T = 30 °C. (**b**) Effect of pH on the removal of MB dyes at 100 ppm concentration, T = 30 °C and 60 min contact time.

The effect of contact time on the removal of MB dye was investigated by varying the duration from 15 to 120 min with an increment of 15 ^o^C. To eliminate the influence of acidity or alkalinity, the liquid medium was maintained at a neutral pH. An initial dye concentration of 100 ppm and 30 ^o^C was used for the analysis. The results, illustrated in Fig. 8**(a)** as a scatter plot showing removal efficiency against contact time, reveal a distinct trend. The removal efficiency increased gradually during the initial phase, reaching 45 min, then exhibited a significant rise to 86% at 60 min before stabilising. This pattern can be divided into three distinct stages. In the first stage (15–45 min), removal efficiency increases as dye molecules begin to occupy the active sites on the biochar surface. The second stage (45–60 min), marked by a sharp rise, likely corresponds to rapid adsorption of dye molecules onto the available active sites. Finally, in the third stage, removal efficiency plateaus, indicating that most of the dye molecules have been adsorbed, leading to a reduced concentration gradient and equilibrium within the system. Beyond this point, no significant increase in removal efficiency is observed. Based on these findings, the optimal contact time for effective dye removal is determined to be 60 min. This conclusion aligns with observations by Singh et al. (2021) on MB removal using biochar derived from *Acacia nilotica*. Their study also noted a steady increase in adsorption capacity up to the equilibrium point, after which the system stabilised, consistent with the behaviour observed in this research^90^.

#### Influence of pH on methylene blue dye removal

The influence of pH on the removal efficiency of methylene blue dye was evaluated by varying the pH of the liquid medium from 2 (acidic) to 12 (basic). The initial dye concentration was maintained at 100 ppm, which was identified as optimal, while the experiment was conducted at 30 °C, ensuring efficient conditions. The contact time for each trial was set to be 60 min. The results (Fig. 8**((b)**) indicates that the removal efficiency reached its maximum value of 86% at pH 7. Notably, the increase in removal efficiency from pH 6 to pH 7 was more significant (12%) compared to other pH intervals. The general trend observed was an increase in removal efficiency with rising pH. The charge dynamics of the biochar surface can explain this behaviour. Under acidic conditions, the biochar surface is dominated by positively charged ions, which repel the cationic dye molecules, resulting in lower adsorption^91^. As the pH increases, the positive surface charge diminishes, allowing negatively charged sites to dominate, thereby enhancing electrostatic attraction and improving dye adsorption. Consequently, the optimal pH for methylene blue removal was determined to be neutral (pH 7). However, a further increase in pH from 7 to 12 showed a decrease in removal efficiency. Increasing the pH from 7 to 12 reduces adsorption efficiency due to excessive hydroxide ions (OH⁻) in the medium, which compete with dye molecules for active adsorption sites on the biochar surface. This competition weakens the electrostatic interactions, leading to decreased dye removal efficiency at highly alkaline conditions. These findings align with the research by Yu et al. (2021), which investigated the effect of pH on methylene blue removal using wet-torrefied microalgal biochar. Their study determined the optimal pH range for methylene blue adsorption to be 2–8, reinforcing the observations reported in this study^85^.

## Adsorption modelling

### Adsorption kinetics of MB

**Fig. 9:**
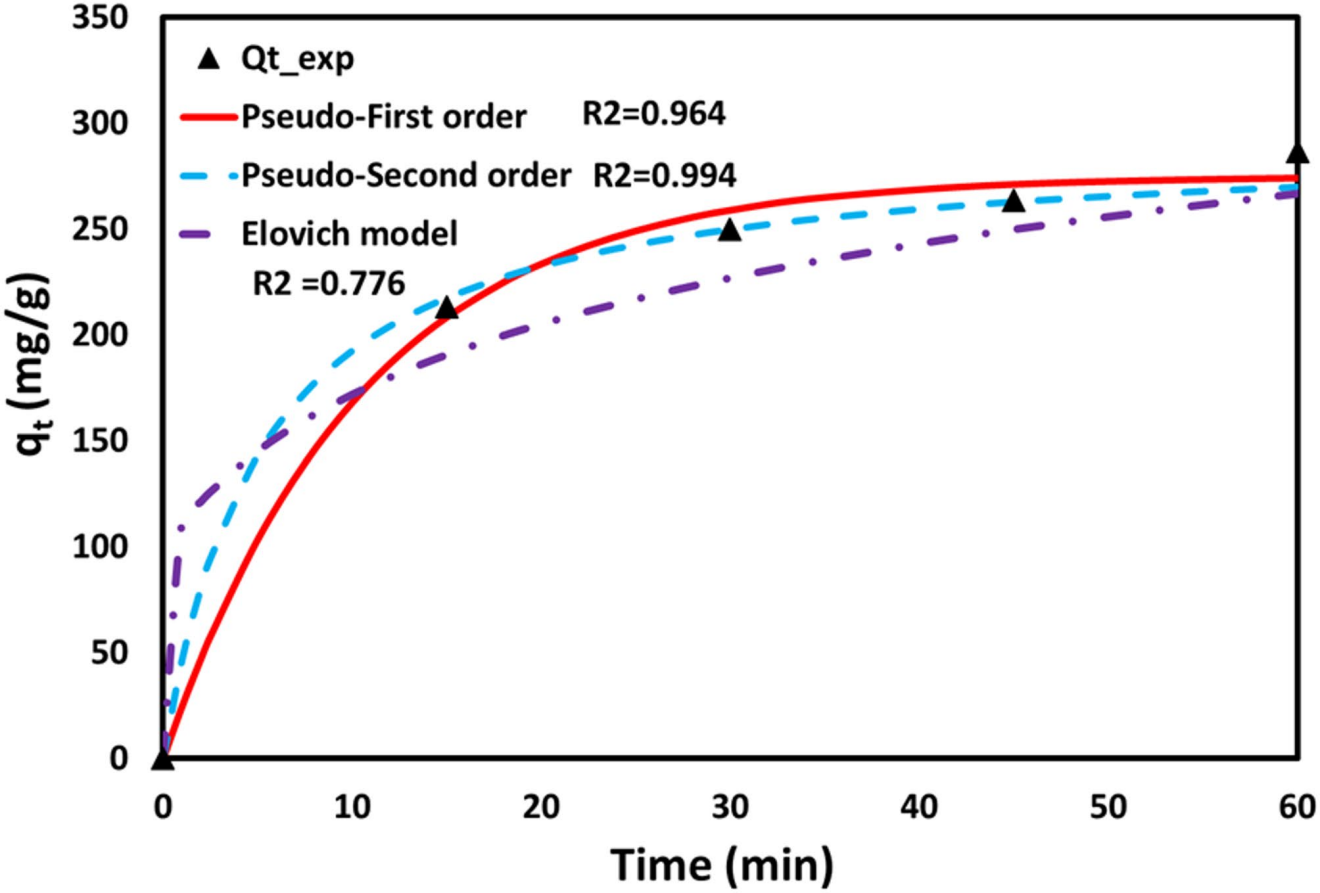
The kinetics model plots of the adsorption experiment of MB pH 7, t = 60 min, C_0_ = 100 ppm, T = 30 °C.

The adsorption performance for removing MB dye was assessed using pseudo-first-order (PSO1), pseudo-second-order (PSO2) and Elovich kinetic models (EKM). The pseudo-first-order model indicates that the adsorption rate is dependent on the available active sites on the adsorbent surface (Fig. 9). The pseudo-second-order model suggests that chemisorption controls the adsorption process between biochar and dye molecules. The pseudo-second-order model suggests chemisorption dominates the adsorption process because it assumes that adsorption occurs through chemical bonding between the adsorbent and adsorbate. Further, the Elovich model suggests that the adsorption mechanism between the biochar and dye molecule involves heterogeneous chemisorption. This model accounts for the involvement of active sites and reflects a rate-limiting step, indicating that the formation of chemical bonds 20 primarily governs the adsorption mechanism. Table 6 presents the parameters of each kinetic model, along with the R² value, which indicates the model that best fits the data. The pseudo-second-order model best fits the experimental data, with an R² value of 0.9940, indicating chemisorption as the primary mechanism. Naushad et al. (2019) investigated the adsorption of cationic dyes using arginine-modified activated carbon. They found that the adsorption mechanism followed chemisorption, as the pseudo-second-order model provided the best fit^92^. Thillainayagam et al. (2023) studies on the removal of methylene blue dye using microalgae biochar in batch and column systems have shown promising results. Microalgae biochar effectively adsorbs MB due to its high surface area and functional groups. Furthermore, the batch and column studies demonstrate efficient dye removal, with improved adsorption capacities observed under optimised conditions^21^.

**Table 6:** Kinetics parameters of adsorption kinetic models.

Kinetics model	Constants	SSE	*R* ^2^	MAPE	NAPE
Pseudo-first order model	q_e_=275 mg/g k1 = 0.0945 min^− 1^	380.29	0.964	2.02	1.81
Pseudo-second order model	qe = 293.6 mg/g k1 = 6.5E-4 min^− 1^ (g/(mg.min))	61.87	0.994	2.09	1.72
Elovich model	qe = 297 (mg/(g.min)) β = 0.0156 (g/(mg))	4083.44	0.776	7.70	6.06

SSE = Sum of Squared Errors, MAPE = Mean Absolute Percentage Error, NAPE = Nonlinear Average Percentage Error.

### Adsorption isotherm

**Fig. 10:**
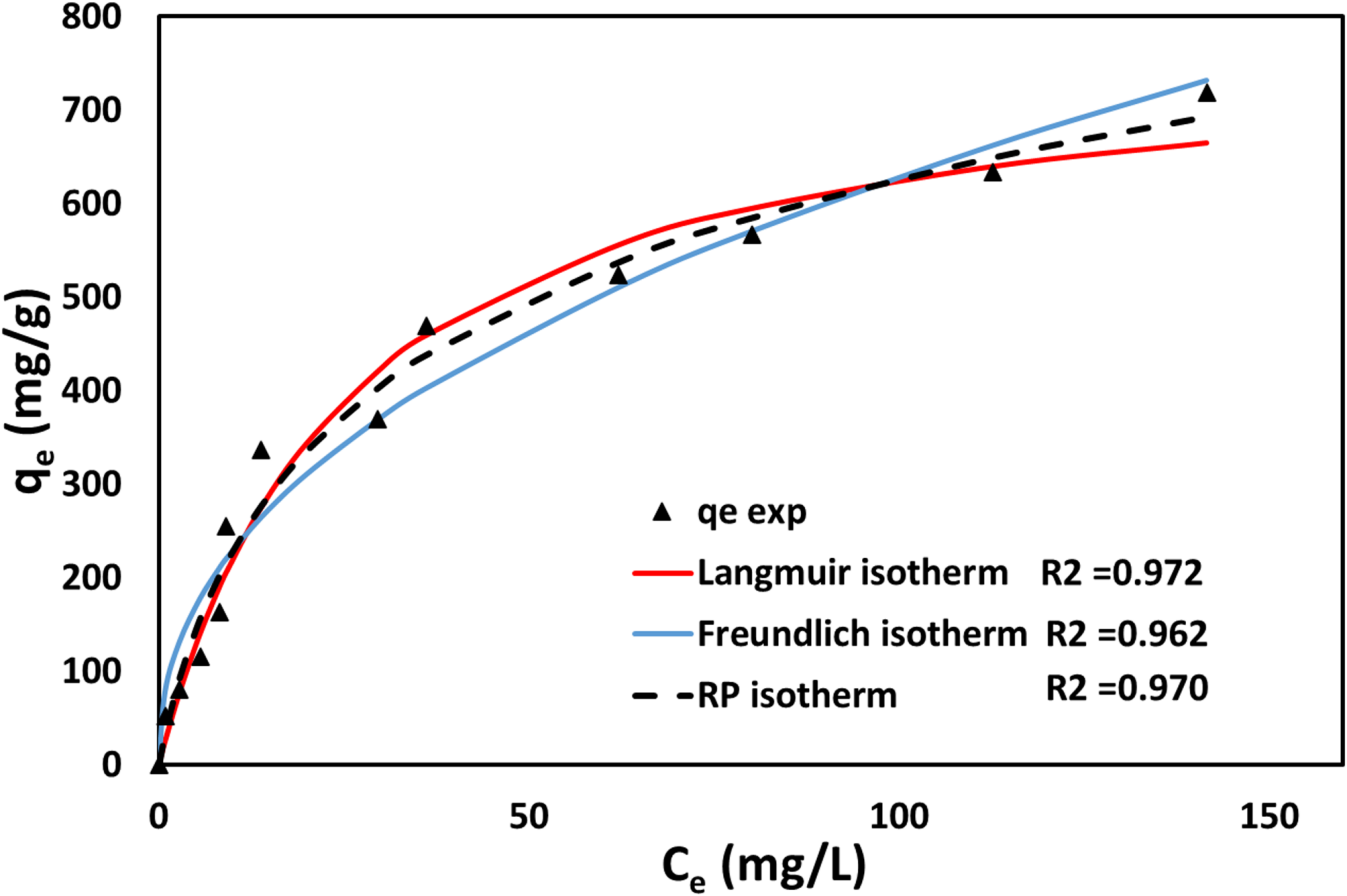
Adsorption Isotherm plot of the experiment, at pH 7, t = 60 min, C_0_ = 100 ppm, T = 30 ^o^C.

The adsorption performance of biochar for removing MB dye was assessed using widely applied isotherm models Langmuir and Freundlich. The Langmuir isotherm assumes a homogeneous adsorbent surface, where dye molecules bind to uniform adsorption sites, forming a monolayer. Conversely, the Freundlich isotherm describes a heterogeneous adsorbent surface, allowing multilayer adsorption on uneven adsorption sites (Fig. 10). The value of R^2^ was found to be 0.9950 and 0.9750 for Langmuir and Freundlich. The model parameters and R² values for the isotherms show that the Langmuir model offers a better fit for the experimental data compared to the Freundlich model. From the results, it was noticed that Langmuir isotherm is more closely followed by adsorption. This implies that monolayer adsorption on a homogeneous surface predominates over multilayer adsorption on a heterogeneous surface. Song et al. (2013) studied the adsorption of phenol and MB using activated carbon derived from rice husk and NAOH treated. They stated that adsorption took place via the Langmuir monolayer adsorption model. This indicates that the adsorption process involves multilayer adsorption on heterogeneous surfaces^93^. Dawood et al. (2016) studied the adsorption of MB dye from an aqueous solution using biochar derived from Eucalyptus sheathiana bark. The adsorption isotherm model parameters of MB’s adsorption are represented in Table 7. As R^2^ values were for the isotherms study confirmed that the adsorption process occurred via the Langmuir monolayer adsorption model^94^.

**Table 7:** Adsorption isotherm model parameters of adsorption of MB.

Isotherm model	Parameters	*R* ^2^	MAPE	NAPE
Freundlich	K_F_=84.027 L/mg *n* = 2.29	0.962	19.846	6.670
Redlich-Peterson model	K_RP_ = 44.647 L/mg α = 0.1455 L/mg β = 0.812	0.970	13.243	6.730
Langmuir	Q_m_ =785.34 mg/L K_a_= 0.039 L/mg	0.972	12.730	6.532

### Proposed mechanism of adsorption

**Fig. 11:**
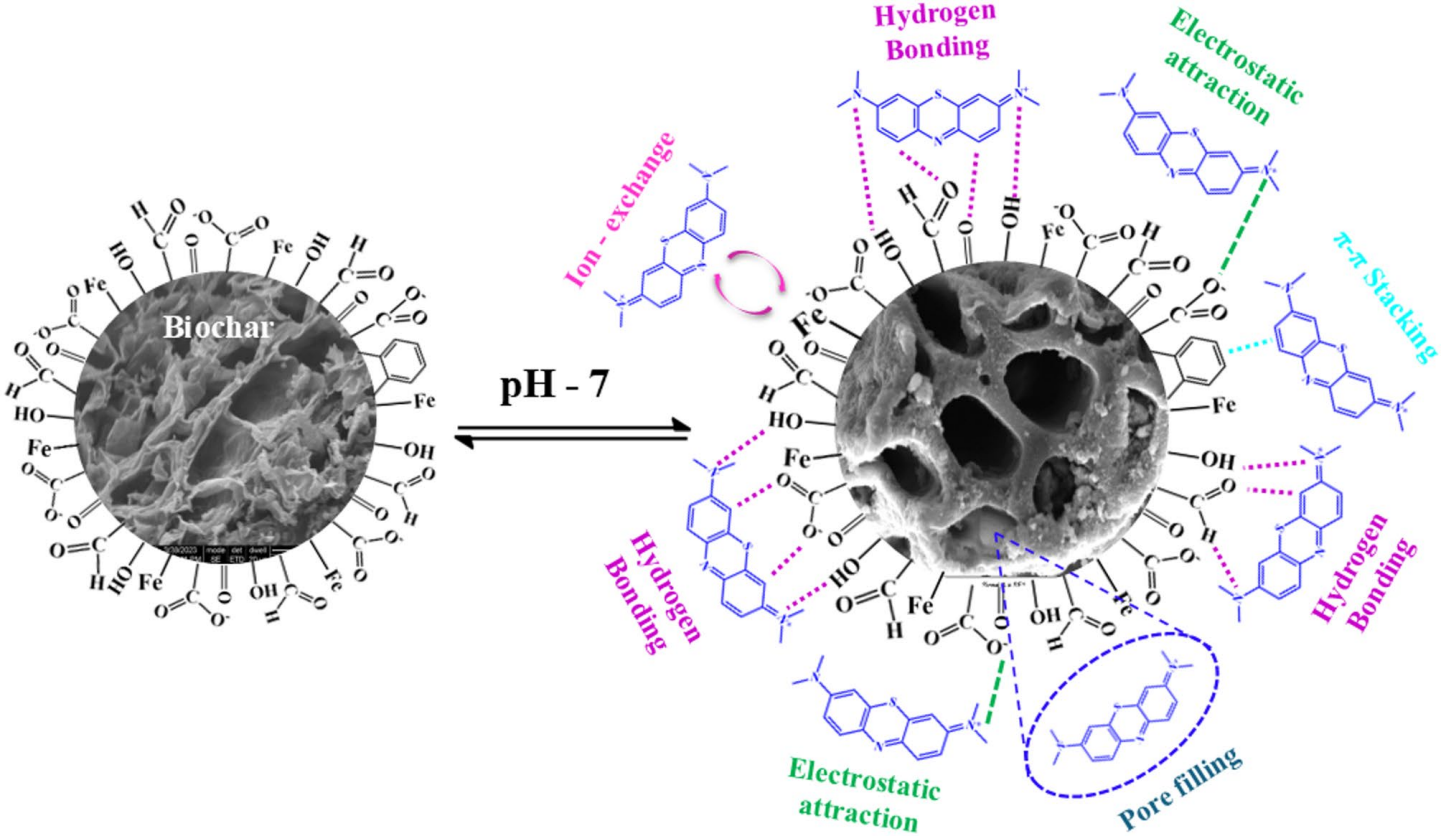
The adsorption mechanism for methylene blue (MB) dye onto biochar.

The interactions between metal particles and carbon materials, electrostatic interaction, hydrogen contact, and π-π interaction are typical explanations for the potential mechanisms of adsorption between metal-doped porous biochars and organic contaminants (adsorbates) (Fig. 11)^95^. Additionally, pore filling is one of the primary mechanisms in adsorbates since materials are highly porous. Anionic dyes and carbon materials doped with cationic metals are drawn to one another by electrostatic forces to create chemical bonds^95^. The adsorption process of methylene blue (MB) dye onto biochar involves many interactions, hence increasing the efficiency of dye removal. Electrostatic attraction is a key factor in interactions between the positively charged MB molecules and the negatively charged functional groups (-COO⁻, -OH) on the biochar’s surface^96^. Additionally, MB’s aromatic rings overlap with biochar’s π-electron-rich carbon structure, creating π-π stacking interactions that support strong adsorption. The formation of hydrogen bonds between the hydroxyl (-OH) and carboxyl (-COOH) groups of biochar and the MB amine (-NH₂) and sulfonate (-SO₃) groups is another mechanism that enhances adhesion^96^. Furthermore, surface complexation takes place in magnetic biochar, where FeO₄ nanoparticles work in tandem with MB molecules to enhance adsorption, hence improving separation and reusability. These combined interactions make biochar a suitable adsorbent for wastewater treatment, aiding in the firm and efficient removal of MB dye from aqueous solutions^13^. Zhang et al. (2013) studied the adsorption of carbaryl and atrazine using pig manure-derived biochars. The adsorption results show an effective removal of carbaryl and atrazine, with higher efficiency in biochars having greater surface area, porosity, and oxygen-containing functional groups. The key functional groups involved are hydroxyl (-OH), carbonyl (C = O), and carboxyl (-COOH), which enhance interactions through hydrogen bonding, π–π stacking, and electrostatic attractions, promoting both adsorption and catalytic hydrolysis processes^97^. Furthermore, the aromatic molecule MB can interact π-π with the aromatic compounds present in biochar^96^. Additionally, the cationic dye MB’s affinity for the negatively charged biochar surface causes electrostatic adsorption^96^. Foo et al. (2012) show that pineapple peel-based activated carbons prepared via microwave-assisted KOH and K₂CO₃ activation exhibit high surface area and well-developed porous structures, enhancing adsorption capacity. Further, the key functional groups responsible for adsorption include hydroxyl (-OH), carboxyl (-COOH), and carbonyl (C = O), facilitating pollutant binding through hydrogen bonding and electrostatic interactions^98^. Electrostatic attraction is the force of attraction between oppositely charged objects, where positive charges attract negative charges and vice versa. This attraction is a fundamental force in physics and chemistry, playing a key role in phenomena like ionic bonding and the behaviour of charged particles. In methylene blue solution, it is present in cationic form (MB^+^), this cation can attract the present anions in the Biochar surface and form a bond. This is one of the reasons for adsorption^99^. Furthermore, hydrogen bonding plays a role in the interaction between MB and Biochar. The nitrogen atoms in the methylene blue molecule can form hydrogen bonds with oxygen-containing functional groups, such as OH or CO, present on the surface of the biochar. Additionally, the hydrogen atom of the aromatic ring can form a hydrogen bond with the oxygen atom on the surface of the biochar. So, hydrogen bonding is one of the reasons for adsorption^26^. One of the main reasons for adsorption is pore-filling. Biochar has a lot of pores on its outer surface. When the methylene blue molecule encounters biochar, it can enter the pores present on the surface of the biochar^6^. The contribution of each mechanism depends only on the properties of the biochar, the characteristics of methylene blue, and the chemistry of the solution.

### Desorption studies

**Fig. 12:**
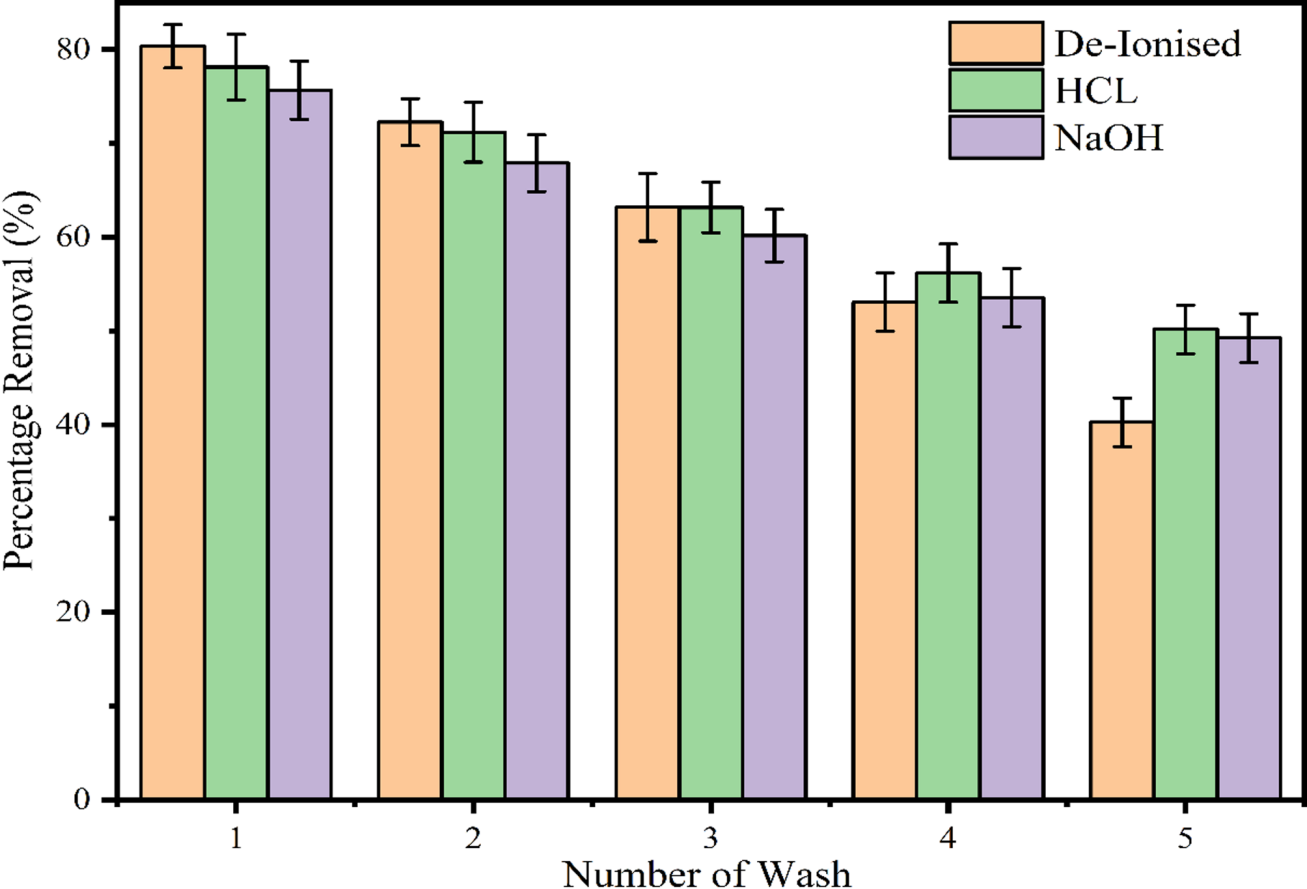
Desorption study bio-adsorbents at t = 60 min, C_0_ = 100 ppm, T = 30 ^o^C.

The selection of an adsorbent is a critical factor in determining the overall efficiency and reusability of an adsorption process. The choice of adsorbent is generally based on two key criteria: its effectiveness in removing the target compound and the ease of recovering the adsorbed compound. Desorption studies are essential to gain a comprehensive understanding of the most suitable method for retrieving methylene blue dye from biochar. The deionized water (H₂O), sodium hydroxide (NaOH), and hydrochloric acid (HCl) were selected as elutants to facilitate the release of methylene blue from biochar. The spent biochar was subjected to five consecutive washes with each elutant, and the desorption efficiency was calculated. After completing the experiments, the results were compiled and graphically represented in Fig. 12. The data clearly indicate that desorption efficiency declines progressively with an increasing number of cycles for all three elutants. This reduction could be attributed to the concentration gradient that develops over successive washes, making the desorption process less effective. Among the three pollutants, NaOH achieved desorption efficiencies of 80.35, 27.26, 63.17, 53.05, and 40.25% over the first, second, third, fourth and fifth washes, respectively. Similarly, H₂O exhibited recovery rates of 78.12, 71.18, 63.16, 46.16 and 50.16% across the five cycles. HCl demonstrated desorption efficiency of 75.66, 67.88, 60.16, 53.55 and 49.23% for the first, second, third, fourth and fifth washes, respectively. A comparative analysis of the results reveals that NaOH is the most effective elutant for desorbing methylene blue from biochar. This superior performance may be attributed to the electrostatic interactions between NaOH and the dye molecules. NaOH-treated adsorbents exhibit maximum adsorption due to enhanced surface area, increased pore formation, and functional group modification. Alkali treatment introduces more hydroxyl and carboxyl groups, improving electrostatic interactions and chemical bonding with target molecules. In contrast, HCl may cause surface erosion, while deionized water lacks activation effects, reducing adsorption capacity. Jayaseelan et al. (2022) studied the desorption efficiency of various pollutants using biochar derived from *Chlorella vulgaris microalgal* biomass for the adsorption of Remazol Blue, malachite green, and *rhodamine B* dyes from wastewater also demonstrated that biochar can be reused for up to four cycles. This finding supports the potential for biochar regeneration and reuse in dye removal applications^100^. Thillainayagam et al. (2023) studied the adsorption of MB dyes using biochar derived from microalgae. The desorption studies assess biochar’s reusability by evaluating elutants like HCl, NaOH, and H₂O. Results indicate decreasing desorption efficiency with repeated cycles due to concentration gradients. HCl exhibited the highest efficiency, likely due to electrostatic interactions. Optimising desorption enhances biochar’s sustainability for wastewater treatment applications^21^.

## Conclusions

The current work used slow pyrolysis to synthesise and characterise magnetic biochar derived from ANH. The porosity, TGA, proximate and ultimate analysis, BET surface area, and other factors were used to characterise the biomass and biochar. Additionally, mineralogy was investigated using XRD analysis, and surface microscopic detection was performed using SEM. The produced biochar was treated using H_2_SO_4_ at 1, 5, 7 and 9 M to improve the biochar surface characteristics. The characterisation of ANH verified that 49 wt% carbon and 78 wt% volatile matter. Additionally, biochar generated at 800 ^o^C (ANHB800) was physicochemically characterised, which revealed that the quality was higher than biochar generated at 600 ^o^C. The acid treatment results confirmed 5M H_2_SO_4_ is a promising reduction in ash content and an increase in BET surface area. The absorption study of MB dye confirmed 85.47% removal at 0.3 g/l dose, 100 ppm concentration, 30 ^o^C, 60 min contact time and 7 pH. The adsorption kinetics results confirmed that the pseudo-second-order model best fits the experimental data, having an R^2^ value of 0.994. Furthermore, the adsorption isotherms study was best fit by the Langmuir adsorption isotherm model conforming monolayer adsorption on the biochar surface.

## Data Availability

The datasets generated during and/or analyzed during the current study are available from the corresponding author upon reasonable request.
